# Apoptosis and tissue thinning contribute to symmetric cell division in the developing mouse epidermis in a nonautonomous way

**DOI:** 10.1371/journal.pbio.3001756

**Published:** 2022-08-15

**Authors:** Arad Soffer, Adnan Mahly, Krishnanand Padmanabhan, Jonathan Cohen, Orit Adir, Eidan Loushi, Yaron Fuchs, Scott E. Williams, Chen Luxenburg

**Affiliations:** 1 Department of Cell and Developmental Biology, Sackler Faculty of Medicine, Tel Aviv University, Tel Aviv, Israel; 2 Department of Biology, Technion—Israel Institute of Technology, Haifa, Israel; 3 Departments of Pathology & Laboratory Medicine and Biology, Lineberger Comprehensive Cancer Center, The University of North Carolina, Chapel Hill, North Carolina, United States of America; Institut Curie, FRANCE

## Abstract

Mitotic spindle orientation (SO) is a conserved mechanism that governs cell fate and tissue morphogenesis. In the developing epidermis, a balance between self-renewing symmetric divisions and differentiative asymmetric divisions is necessary for normal development. While the cellular machinery that executes SO is well characterized, the extrinsic cues that guide it are poorly understood. Here, we identified the basal cell adhesion molecule (BCAM), a β1 integrin coreceptor, as a novel regulator of epidermal morphogenesis. In utero RNAi-mediated depletion of *Bcam* in the mouse embryo did not hinder β1 integrin distribution or cell adhesion and polarity. However, *Bcam* depletion promoted apoptosis, thinning of the epidermis, and symmetric cell division, and the defects were reversed by concomitant overexpression of the apoptosis inhibitor *Xiap*. Moreover, in mosaic epidermis, depletion of *Bcam* or *Xiap* induced symmetric divisions in neighboring wild-type cells. These results identify apoptosis and epidermal architecture as extrinsic cues that guide SO in the developing epidermis.

## Abstract 2

Mitotic spindle orientation and symmetric versus asymmetric cell division are conserved mechanisms that govern cell fate and tissue morphogenesis. This study shows that apoptosis functions as an external cue that enhances symmetric cell division in the developing epidermis.

## Introduction

Spindle orientation (SO) is a highly conserved and tightly regulated process that plays a key role in cell fate determination, tissue morphogenesis, and homeostasis (reviewed in [1–4]). In the developing epidermis, basal layer stem cells/progenitors orient their spindles either parallel or perpendicular to the basement membrane (BM). After parallel (symmetric) division, the two daughter cells remain in the basal layer and may proliferate; in contrast, following perpendicular (asymmetric) division, one daughter cell remains in the basal layer while the other daughter cell becomes suprabasal and begins to differentiate (reviewed in [5–9]).

Studies in the developing epidermis showed that SO is essential for survival and barrier formation [10–12] and additionally plays a role in cell competition, a selection process that optimizes epidermal development [13], and the specification of hair follicle stem cells [14]. In the adult epidermis, SO protects the tissue against oncogene-induced hyperproliferation [15], regulates hair follicle morphogenesis [10,14], and controls the fate of hair follicle stem cells [16] and matrix cells [10].

The molecular machinery that executes SO is highly conserved [17,18] and has been extensively characterized in several model systems including the mammalian epidermis. Several proteins have been shown to play crucial roles in SO, including the apical polarity complex Par3–Par6–aPKC, which interacts with LGN–Gα(i)–NuMA–dynein–dynactin, a complex that directs positioning of the spindle [10,11,12,16,19,20]. Adhesion proteins also play an important role in SO. The adherens junction proteins vinculin, α-catenin, and afadin are essential for telophase reorientation and oriented cell division fidelity [21]. Similarly, β1 integrin is also essential for SO, presumably through its function in the maintenance of cell–extracellular matrix adhesion [19].

Basal cell adhesion molecule (BCAM, also known as Lu/Lutheran blood group) is a member of the immunoglobulin superfamily and functions as a β1 integrin coreceptor by modulating its interaction with the BM protein laminin [22–24]. BCAM is a transmembrane glycoprotein that was first identified in red blood cells; however, it is highly expressed in epithelial cells of most organs [22,23,25]. Deletion of *Bcam* does not hinder mouse viability or fertility; however, in the kidney, the BM was abnormally thick and capillary number was decreased, while in the intestine, BM appeared normal but the smooth muscle coat was abnormally thick and disorganized [26].

Although β1 integrin is a well-studied major regulator of epidermal biology [27–32], the role of BCAM in epidermal development is unknown. Therefore, in the present study, we investigated the roles of BCAM in epidermal development in the mouse. Unexpectedly, we found that BCAM activity is dispensable for β1 integrin distribution, cell adhesion, and BM assembly; however, BCAM was shown to play an essential role in cell survival, tissue architecture, and balanced SO in the epidermis.

## Results

### BCAM depletion does not alter β1 integrin distribution or activity in the developing mouse epidermis

**Fig 1:**
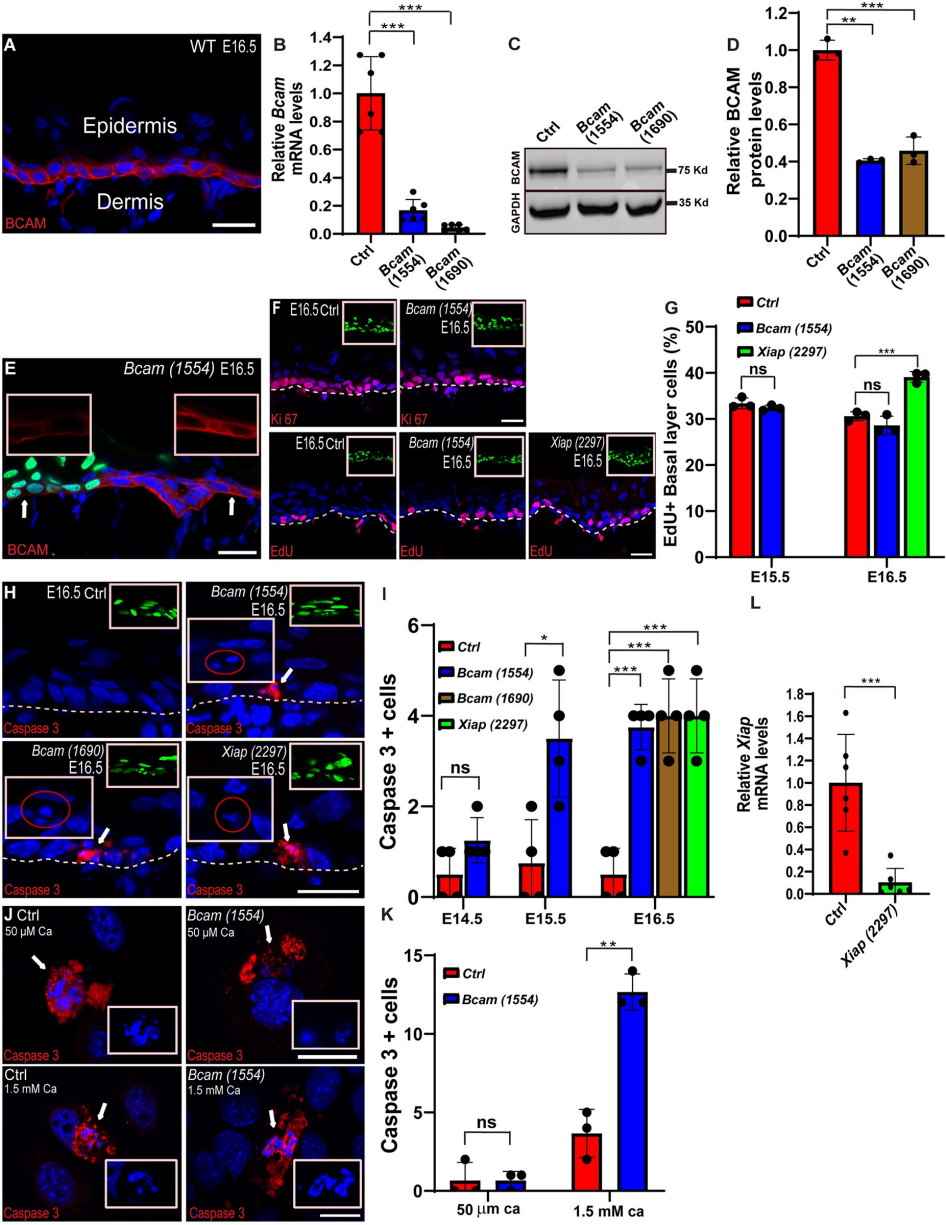
*Bcam* depletion induces apoptosis in the developing epidermis. (A) Sagittal views of 10-μm sections of dorsal skin from E16.5 CD1 mouse embryos immunostained for BCAM (red). (B) RT-PCR analysis of *Bcam* mRNA in primary mouse keratinocytes transduced with scrambled shRNA (Ctrl) or the two *Bcam*-specific shRNAs *1554* and *1690*. Data are the mean ± SD of *n =* 6 experiments per condition from 3 independent experiments. ***P = 3 × 10^−4^ for control vs. *Bcam*-*1554* and ***P = 3.2 × 10^−4^ for control vs. *Bcam*-*1690* by unpaired *t* test. (C) Western blot analysis of BCAM protein expression in primary mouse keratinocytes transduced with *shScr* (Ctrl), *Bcam*-*1554*, or *Bcam*-*1690* shRNAs. GAPDH was probed as a loading control. (D) Quantification western blot analysis shown in (C). Data are the mean ± SD of *n =* 3 blots **P = 2 × 10^−3^ for control vs. *Bcam*-*1554*; ***P = 8 × 10^−4^ for control vs. *Bcam*-*1690* by unpaired *t* test. (E) Sagittal views of 10-μm sections of dorsal skin from *Bcam*-*1554* KD E16.5 mosaic tissue immunostained for BCAM (red). (F) Sagittal views of 10-μm sections of dorsal skin from control, *Bcam*-*1554* and *Xiap-2297* KD E16.5 embryos immunostained for the cell proliferation marker Ki67 (red, upper panel) or pulsed for 2 h with EdU (red, lower panel). (G) Quantification of EdU+ cells from the images shown in (F). Data are the mean ± SD of *n =* 3 embryos per condition. E15.5, not significant for control vs. *Bcam*-*1554* (*P* = 0.2754); E16.5, ***P = 8 × 10^−4^ for control vs. *Xiap-2297*; not significant for control vs. *Bcam*-*1554* (*P* = 0.223) by unpaired *t* test. (H) Sagittal views of 10-μm sections of dorsal skin from control, *Bcam*-*1554*, *Bcam*-*1690*, and *Xiap*-*2297* KD E16.5 embryos immunostained for active caspase 3 (red). Arrows indicate active caspase 3+ cells. Large boxes/ovals show magnification of fragmented nuclei in active caspase 3+ cells. (I) Quantification of active caspase 3+ cells from the data shown in (H and S3 and S4 Figs). Data are the mean ± SD of *n =* 4 embryos per condition. E14.5, not significant for control vs. *Bcam*-*1554* (*P* = 0.0981); E15.5, *P = 1.6 × 10^−2^ for control vs. *Bcam*-*1554;* E16.5, ***P = 2 × 10^−4^ for control vs. *Bcam*-*1554*, ***P = 7 × 10^−4^ for control vs. *Bcam*-*1690*, ***P = 7 × 10^−4^ for control vs. *Xiap*-*2297* by unpaired *t* test. (J) *shScr*- (Ctrl) and *shBcam-1554*-transduced primary mouse keratinocytes were cultured in low- (50 μM) or high-calcium (1.5 mM) media and then immunolabeled for active caspase 3. (K) Quantification of active caspase 3+ cells from the data shown in (J). Data are the mean ± SD of *n =* 3 experiments. Low calcium, not significant (*P* > 0.999); high calcium, **P = 1 × 10^−3^ for control vs. *Bcam-1554* by unpaired *t* test. (L) RT-PCR analysis of *Xiap* mRNA in primary mouse keratinocytes transduced with scrambled shRNA (*Ctrl*) or *Xiap*-specific shRNA (*2297*). Data are the mean ± SD of *n =* 6 experiments per condition from 3 independent experiments. ***P = 3 × 10^−3^ for control vs. *Xiap*-*2297* by unpaired *t* test. The data underlying all the charts in the figure are included in S1 Data. Nuclei were stained with DAPI (blue). Dotted lines indicate the dermal–epidermal border, and upper right insets show the transduced cells (H2B-GFP+). Scale bars = 20 μm. BCAM, basal cell adhesion molecule; RT-PCR, reverse transcription PCR.

We began our investigation of the potential role of BCAM in β1 integrin-dependent and/or β1 integrin-independent functions in epidermal development by examining BCAM localization in mouse embryos. Immunofluorescent staining of dorsal skin from day 16.5 embryos (E16.5) of wild-type CD1 mice revealed high levels of BCAM throughout the cortex of basal layer cells, similar to what has been reported for β1 integrin expression (Figs 1A and S1).

To facilitate the analysis of BCAM function, we screened several *Bcam*-specific short hairpin RNAs (shRNAs) and identified two, *Bcam*-*1554* and *Bcam*-*1690*, which depleted *Bcam* mRNA levels in primary mouse keratinocytes (1°MKs) by 83 ± 7.7% and 96 ± 2.3%, respectively, compared with control scrambled shRNA (*shScr*) (Fig 1B). Western blot analysis confirmed the mRNA results and showed that BCAM protein levels were similarly depleted in *shBcam*-expressing compared with control 1°MKs (Fig 1C and 1D).

To deplete *Bcam* during epidermal development, the amniotic sacs of E9 wild-type mouse embryos were injected in utero with lentiviruses encoding *Bcam*-*1554*, *Bcam*-*1690*, or *shScr* together with a GFP-tagged histone 2B reporter (H2B-GFP) to identify successfully transduced cells [33]. Immunostaining with BCAM antibody in mosaic tissue confirmed the depletion of BCAM in H2B-GFP+ cells in the dorsal skin of E16.5 embryos (Fig 1E).

We first asked whether BCAM depletion affects the localization of β1 integrin in the dorsal skin of E16.5 embryos, but no differences were detected in β1 integrin localization between control and *Bcam* knockdown (KD) embryos; β1 integrin was present throughout the cortex of basal layer cells with the highest levels observed in the basal region of the cell juxtaposed with the BM (S1 Fig). Moreover, immunostaining with a 9EG7-specific antibody, which recognizes an epitope unique to active β1 integrin [34], revealed comparable distribution and expression levels (fluorescence intensity) in the control and *Bcam* KD epidermis (S1 Fig). Because β1 integrin is essential for skin BM assembly [35,36] and BCAM itself directly binds to the BM protein laminin α5 [22–24,37], we next examined BM organization by immunostaining the dorsal skin of control and *Bcam* KD E16.5 embryos for laminin γ1 (represented by laminins 511 and 521 in the skin, which both contain α5 laminin chain), laminin 332, and nidogen, all of which are major components of the skin BM [38,39]. Each protein was detected as a thin line between the epidermis and the dermis in both control and *Bcam* KD epidermis (S1 Fig). Together, these data indicate that BCAM expression is not necessary for the distribution or activity of β1 integrin, or for cell adhesion or BM organization in the developing epidermis.

### BCAM depletion does not hinder cell–extracellular matrix adhesion in cultured keratinocytes

Focal adhesions are integrin-based structures that mediate cell adhesion to the extracellular matrix [40]. β1 integrin is a major regulator of focal adhesions in many cell types including cultured 1°MKs [28,35]. To examine whether BCAM activity alters β1 integrin levels, activity, and focal adhesion organization, 1°MKs were transduced with lentiviruses encoding *shScr* (control) or *shBcam-1554* and analyzed by confocal microscopy. Immunostaining analysis of β1 integrin and the 9EG7 epitope levels showed that while overall levels of β1 integrin were comparable in control and *Bcam*-depleted cells, the levels of the 9EG7 epitope increased by 23% in *Bcam*-depleted cells (S2 Fig). Immunostaining for the focal adhesion protein paxillin [41,42] detected comparable numbers of focal adhesions; however, average focal adhesion area increased by 8.8% in *Bcam-*depleted cells (S2 Fig). Together, these data indicate that BCAM loss-of-function does not hinder cell–extracellular matrix adhesion in cultured 1°MKs; instead, it results in a modest increase in β1 integrin activity and focal adhesion area.

### BCAM depletion induces apoptosis

We next determined whether the coreceptor function of BCAM is necessary for β1 integrin contribution to epidermal growth, a process that involves cell proliferation, differentiation, senescence, and death [43,44]. To this end, the dorsal skin of control and *Bcam* KD E16.5 embryos was immunostained for the cell proliferation marker Ki67, but no differences in the pattern of Ki67+ cells were observed (Fig 1F). To quantify proliferation, we injected embryos with *shScr* or *Bcam*-*1554* at E9 and then pulsed the pregnant mice on E15.5 and E16.5 for 2 h with the uridine analog EdU (5-ethynyl-2′-deoxyuridine), which incorporates into S-phase cells. Quantification of EdU+ cells in the embryonic dorsal skin sections confirmed a similar level of proliferation in *Bcam*-depleted compared with control epidermis (E15.5: 33.4 ± 1.2%, control and 32.4 ± 0.6%, *Bcam* KD; E16.5: 30.6 ± 0.8%, control and 28.6 ± 1.7%, *Bcam* KD; Fig 1G).

We next examined whether BCAM was required for epidermal differentiation by immunostaining dorsal skin sections of E14.5, E15.5, and E16.5 embryos for the epidermal cell markers keratin 14 (K14, basal layer), K10 (suprabasal layers), and loricrin (granular layer) (S3–S5 Figs). These analyses revealed normal differentiation of *Bcam*-depleted epidermis. Moreover, induction of K10 was detected when *Bcam*-depleted 1°MKs were induced to differentiate by increasing calcium levels in vitro (S2 Fig). Together, these results indicate that BCAM is not required for normal epidermal differentiation.

Although β1 integrin plays a role in cell senescence pathways [45], we did not detect a difference in expression of senescence-associated β-galactosidase [33,46] between the epidermis of control and *Bcam* KD E16.5 embryos (S5 Fig), suggesting that BCAM does not contribute to this function. In striking contrast, we identified a key role for *Bcam* in cell apoptosis. Notably, immunostaining of E14.5, E15.5, and E16.5 dorsal skin sections for the active, cleaved form of the proapoptotic enzyme caspase 3 revealed very few positive cells in control epidermis (<1 positive cell per dorsal skin section). However, a progressive increase in active caspase 3+ cells was observed in *Bcam* KD epidermis beginning at E15.5, culminating in an approximately 8-fold increase by E16.5 (Figs 1H, 1I, S3, and S4). Since caspase 3 can have nonapoptotic roles in the skin [47], we confirmed that *Bcam*-depleted, active caspase 3+ cells were apoptotic by their nuclear condensation and fragmentation, a classical hallmark of an apoptotic cell [48] (Figs 1H, S3, and S4). Moreover, apoptosis was detected only in infected (GFP+) basal layer cells, confirming this effect on apoptosis is cell autonomous, and is restricted to the proliferative compartments of the epidermis.

To determine whether the defect in apoptosis can be recapitulated in tissue culture, 1°MKs were transduced with lentiviruses encoding *shScr* (control) or *shBcam-1554*, cultured in low- or high-calcium media (50 μM and 1.5 mM, respectively), and immunostained for active caspase 3. In low-calcium media in which keratinocytes cannot form cell–cell junctions and cannot differentiate, very few control or *Bcam*-depleted cells were caspase 3+. However, in high-calcium media, conditions that allow cell–cell adhesion and induce keratinocytes differentiation [49], we detected a 3.5-fold increase in caspase 3+ in *Bcam*-depleted cells (Fig 1J and 1K).

To verify that these findings are indeed indicative of apoptosis in the *Bcam*-depleted epidermis, we performed the same analyses in embryos depleted of x-linked inhibitor of apoptosis (XIAP), which has been shown to function to suppress apoptosis in hair follicle stem cells [50]. E9 embryos were injected in utero with XIAP-targeted shRNA (*shXiap*-*2297*), which we confirmed could effectively deplete *Xiap* mRNA levels (90 ± 12.7% in 1°MKs; Fig 1L), and dorsal skin sections of E16.5 embryos were immunostained for active caspase 3 (Fig 1H). As expected, *Xiap* depletion increased the number of active caspase 3+ cells in the epidermis, consistent with an increase in apoptosis; in fact, the number of apoptotic cells in *Xiap-*depleted epidermis was remarkably similar to that observed in *Bcam*-depleted epidermis (Fig 1I). However, unlike *Bcam*-depleted embryos, E16.5 *Xiap*-depleted epidermis exhibited an approximately 30% increase in EdU incorporation compared with the control epidermis (30.6 ± 0. 8% versus 39.1 ± 1.2%), suggesting that while both BCAM and XIAP have antiapoptotic functions, they have distinct effects on epidermal growth (Fig 1G).

Taken together, these data demonstrate that BCAM is not required for cell proliferation, differentiation, or senescence, but unexpectedly, it is required for cell survival and is a potent inhibitor of apoptosis in the developing epidermis and in cultured keratinocytes.

### BCAM depletion in the developing epidermis causes an increase in symmetric cell division

The growth potential of keratinocytes is tightly regulated by their localization in the stratified tissue; in particular, while basal layer cells have mitotic potential, suprabasal layer cells are generally postmitotic. Two mechanisms control the basal–suprabasal transition in the developing epidermis: delamination [20,51,52] and SO [12,19]. The balance between the two processes changes during development. In the epidermis from E15.5 embryos, delamination levels are high while SO is not accurate, and many cell divisions are oblique. In the epidermis from E16.5 embryos, the number of cells that undergo delamination is down-regulated, and SO becomes bimodal [20,51]. Therefore, we next examined whether BCAM activity affects cell delamination or SO in the developing mouse epidermis.

**Fig 2:**
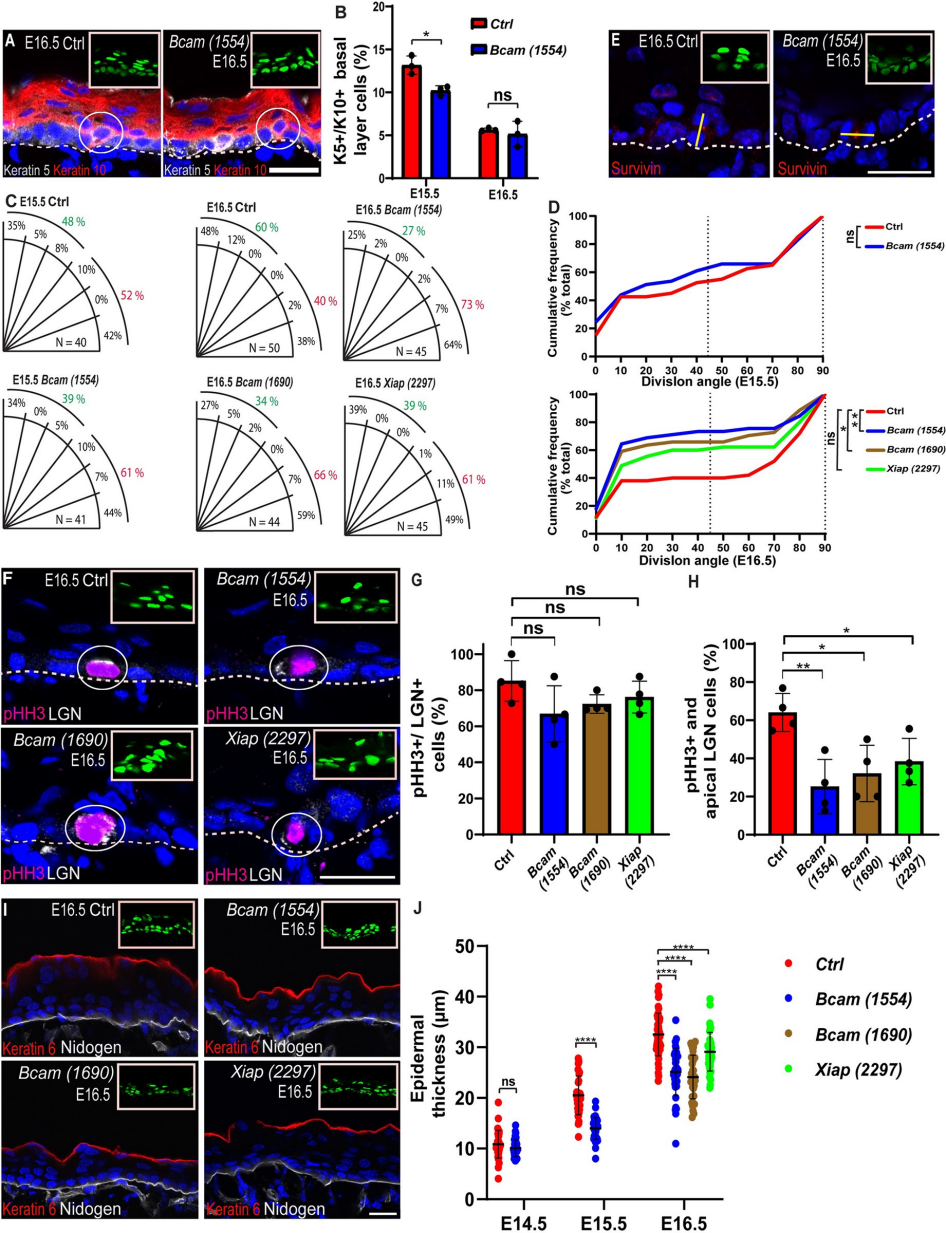
*Bcam* depletion enhances symmetric cell division. (A) Sagittal views of 10-μm sections of dorsal skin from control and *Bcam*-*1554* KD E16.5 embryos coimmunostained for the basal layer marker keratin 5 (white) and the suprabasal layer marker keratin 10 (red). Circles indicate cells positive for both keratin 5 and keratin 10. (B) Quantification of keratin 5/keratin 10 double-positive cells shown in (A and S4 Fig). Data are the mean ± SD of *n =* 3 embryos per condition. E15.5, *P = 1.3 × 10^−2^ for control vs. *Bcam*-*1554*; E16.5, not significant (*P* = 0.598) by unpaired *t* test. (C) Quantification of SO from data shown in (E, S4 and S6 Figs). (D) Same data as in (C), plotted as a cumulative frequency distribution. E15.5, not significant (*P* = 0.6058); E16.5, **P = 9.7 × 10^−3^ for control vs. *Bcam*-*1554*, *P = 4.52 × 10^−2^ for control vs. *Bcam*-*1690*, not significant (*P* = 0.1926) for control vs. *Xiap-2997* by Kolmogorov–Smirnov test. (E) Sagittal views of 10-μm sections of dorsal skin from control and *Bcam*-*1554*, E16.5 embryos immunostained for the cleavage furrow marker survivin (red). Yellow lines show representative axes of division. (F) Sagittal views of 10-μm sections of dorsal skin from E16.5 control, *Bcam*-*1554*, *Bcam 1690*, and *Xiap 2297* coimmunostained for the mitosis marker pHH3 (red) and the SO protein LGN (white). White circles indicate double-positive cells. (G) Quantification of double-positive cells from the data shown in (F). Not significant (*P* = 0.1101) for control vs. *Bcam*-*1554*, (*P* = 0.1024) for control vs. *Bcam*-*1690*, (*P* = 0.2556) for control vs. *Xiap-2297* by unpaired *t* test. (H) Quantification of cells positive for pHH3 and apical crescent LGN from the data shown in (F). **P = 5.5 × 10^−3^ for control vs. *Bcam*-*1554*, *P = 1.14 × 10^−2^ for control vs. *Bcam*-*1690*, *P = 1.8 × 10^−2^ for control vs. *Xiap-2297* by unpaired *t* test. (I) Dorsal skin sections from E16.5 embryos treated as in (F) and immunostained for the periderm marker keratin 6 (red) and the BM marker nidogen (white). (J) Quantification of epidermal thickness from the data shown in (I, S3 and S4 Figs). *n =* 40 microscopic fields from 4 embryos per condition. E14.5, not significant for control vs. *Bcam*-*1554* (*P* = 0.801); E15.5, ***P < 0.0001 for control vs. *Bcam*-*1554;* E16.5, ***P < 0.0001 for control vs. *Bcam*-*1554*, ***P < 0.0001 for control vs. *Bcam*-*1690*, ***P = 2.1 × 10^−4^ for control vs. *Xiap-2297* by unpaired *t* test. Nuclei were stained with DAPI (blue). The data underlying all the charts in the figure are included in S1 Data. Dotted lines indicate the dermal–epidermal border, and upper right insets show the transduced cells (H2B-GFP+). Scale bars = 20 μm. BM, basement membrane; SO, spindle orientation.

To detect delamination, we costained dorsal skin sections from control or *Bcam* KD E15.5 and E16.5 embryos for keratins 5 and 10 and quantified the number of K5+/K10+ double-positive cells within the basal layer [20,52]. In E15.5 *Bcam*-depleted epidermis, we detected an approximately 30% decrease in the number of K5+/K10+ basal layer cells relative to control epidermis, while a comparable number of K5+/K10+ cells were detected in control and *Bcam* KD in E16.5 (E15.5, 13.2 ± 1.1% and 10.2 ± 0.6%; E16.5, 5.65 ± 0.2% and 5.16 ± 1.5% of control and *Bcam* KD basal layer cells, respectively; Figs 2A, 2B, and S4).

Next, we quantified SO by examining the expression of survivin, which stains the cleavage furrow in late-mitotic cells, and then calculated the angle between the two daughter nuclei and the BM [12,53]. As expected at this age, in both control and *Bcam*-depleted E15.5 epidermis, many of the cell divisions were oblique (15° to 75°). Overall, however, in controls, approximately 50% of mitotic spindles were oriented perpendicular to the BM (45° to 90°) and approximately 50% of SO were parallel to the BM (0° to 45°), indicating a balance between symmetric and asymmetric cell division. We detected a modest, but not statistically significant, 17.3% increase in parallel SOs in *Bcam*-depleted epidermis (Figs 2C, 2D, and S4). In E16.5 embryos, the earliest time point when SO becomes bimodal [12,19,20], *Bcam* KD with either *Bcam*-*1554* or *Bcam*-*1690* caused an approximately 70% increase in parallel SOs, such that approximately 70% of total cell divisions were symmetric (Fig 2C–2E). SO analysis in E16.5 *Xiap* KD epidermis showed a more modest 50%, but not statistically significant increase in parallel SOs (Figs 2C, 2D, and S6). This observation is in line with the notion that cell proliferation affects SO [15] and the distinct growth properties of *Bcam*- and *Xiap*-depleted skins.

At the molecular level, perpendicular SO in the interfollicular epidermis requires an apical localization of LGN, a key component of the complex that connects astral microtubules to the apical cortex [12,19]. To better understand the involvement of BCAM in SO, we examined the effects of *Bcam* KD on the abundance and localization of LGN+ cells. The percentage of LGN+ cells within early mitotic cells was determined by costaining for LGN and the early mitotic marker phosphohistone H3 (pHH3). Immunostaining of E16.5 dorsal skin sections showed that the number of LGN+/pHH3+ double-positive cells was about the same in control and *Bcam* KD epidermis (approximately 75%; Fig 2F and 2G). However, whereas approximately 65% of the LGN+/pHH3+ cells in control epidermis had an apical LGN crescent, LGN staining was apical in fewer than 35% of LGN+/pHH3+ cells in *Bcam* KD epidermis (Fig 2F and 2H). Like *Bcam* KDs, a decrease in apical LGN was also observed in *Xiap*-depleted epidermis (Fig 2F–2H). Thus, increased apoptosis leads to reduced apical localization of LGN and decreased perpendicular SO in developing epidermal cells.

LGN depletion or mislocalization has been shown to result in abnormally thin epidermis [12,20], and this defect is likely to be exacerbated by elevated levels of apoptosis, and decreased delamination, as observed in the developing *Bcam* KD epidermis. To determine whether BCAM function affects epidermal thickness, we immunostained dorsal skin from E14.5, E15.5, and E16.5 control and *Bcam* KD embryos for K6, which labels the periderm, a thin layer of cells above the embryonic epidermis, and nidogen, which labels the BM, and measured the epidermal thickness (Figs 2I, 2J, S3, and S4). In E14.5 embryos, comparable epidermal thickness was detected in control and *Bcam*-depleted epidermic (10.9 ± 2.7 μm, 10.7 ± 1.9 μm, respectively). In E15.5 and E16.5 *Bcam*-depleted embryos, the epidermis was approximately 25% thinner than control epidermis (Fig 2I and 2J). In line with the increase in both apoptosis and cellular proliferation in *Xiap* KD embryos, the thickness of E16.5 *Xiap* KD epidermis was 29.1 ± 3.8 μm, between control and *Bcam*-depleted epidermis (Fig 2I and 2J). Collectively, these results are consistent with a crucial role for BCAM in orchestrating the key events that impact on SO and symmetric/asymmetric cell division in the epidermis.

### BCAM is not involved in cell–cell adhesion or apicobasal polarity in the epidermis

Our results thus far identify BCAM’s ability to impact SO. Because cell–cell adhesion and apicobasal polarity have been shown to play important roles in the regulation of SO in the epidermis [16,19,21,53,54], we next investigated whether BCAM expression affects these processes. To this end, we immunostained for the cell adhesion proteins E-cadherin and α-catenin, the apicobasal protein Par3, and the centrosomal protein pericentrin in E16.5 control and *Bcam* KD embryos. However, the localization and intensity of staining of all 4 proteins were comparable between the embryos (S5 Fig). Moreover, normal cell–cell adhesion was detected in E14.5, and E15.5 *Bcam*-depleted embryos (S3 and S4 Figs), and in calcium-shifted cultured keratinocytes (S2 Fig), indicating that BCAM expression is not required for normal epidermal cell–cell adhesion and apicobasal polarity. Similarly, upon examination of basal layer cell shape and mitotic rounding, which regulate SO in the epidermis [55–57], we detected no differences in interphase and mitotic basal layer cell shape in *Bcam*-depleted and control epidermis sections (S7 Fig), indicating that BCAM is unlikely to be involved in these processes.

### BCAM involvement reveals a link between apoptosis and spindle orientation/symmetric cell division

**Fig 3:**
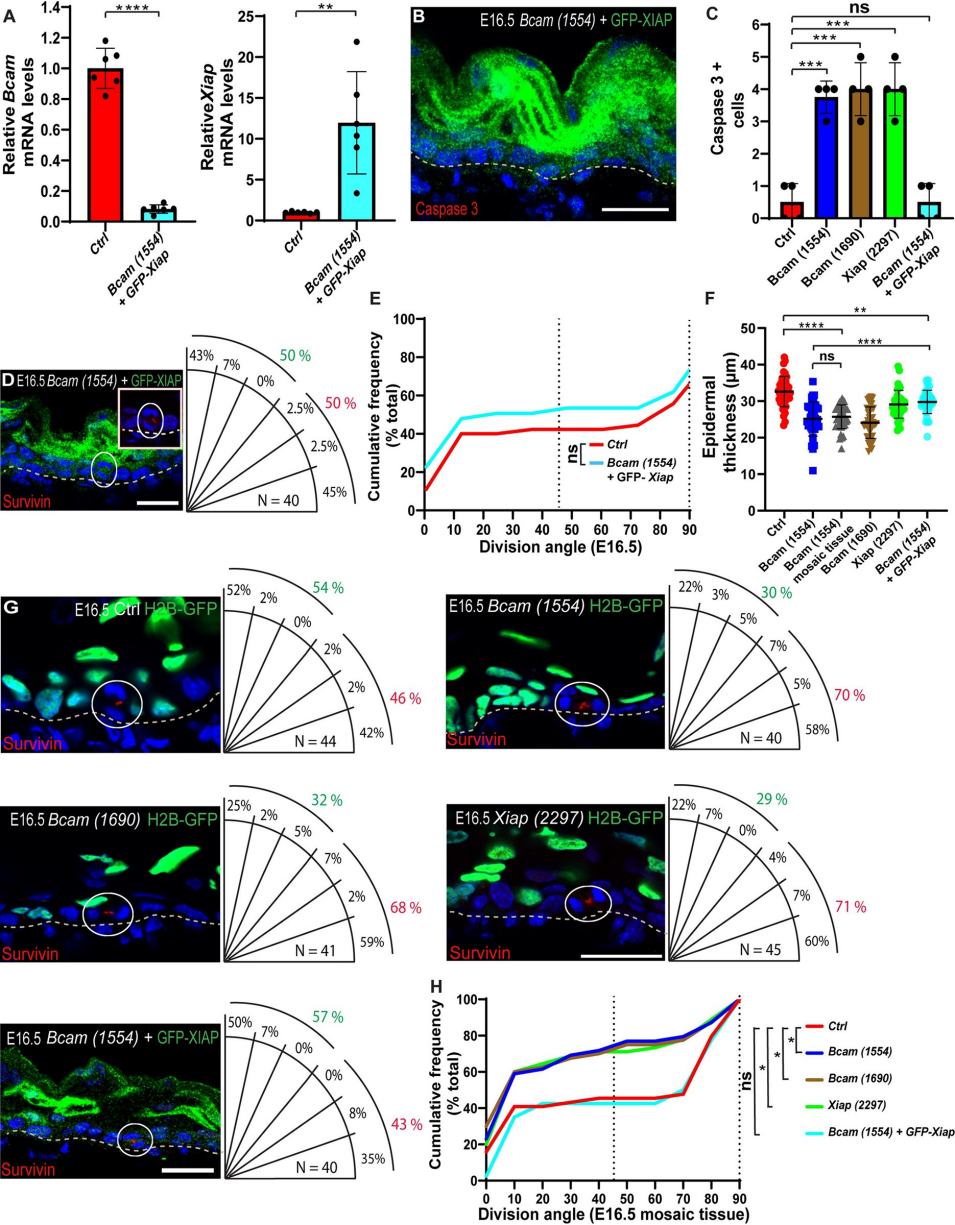
Apoptosis enhances symmetric cell division. (A) RT-PCR analysis of *Bcam* and *Xiap* mRNA in primary mouse keratinocytes transduced with *shScr; H2B-GFP* (Ctrl) or *shBcam*-*1554*; *GFP-Xiap*. Data are the mean ± SD of *n =* 6 experiments per condition from 3 independent experiments. ****P < 10^−4^ for *Bcam* levels in control vs. *Bcam*-*1554* and **P = 7.8 × 10^−3^ for *GFP-Xiap* levels in control vs. *shBcam-1554*;*GFP*-*Xiap* by unpaired *t* test. (B) Sagittal view of a 10-μm section of dorsal skin from a *shBcam-1554*;*GFP*-*Xiap*-transduced E16.5 embryo immunostained for active caspase 3. (C) Quantification of active caspase 3+ cells shown in (B). Quantification of active caspase 3+ cells in Ctrl, *shBcam-1554-*, *shBcam-1554-*, *shXiap-2279-* is shown in Fig 1. Data are the mean ± SD of *n =* 4 embryos per condition. Not significant (*P* = 0.7228) for control vs. *shBcam-1554*;*GFP*-*Xiap* by unpaired *t* test. (D) Sagittal views of 10-μm sections of dorsal skin from *shBcam-1554*;GFP-*Xiap* E16.5 embryos immunostained for the cleavage furrow marker survivin (red). White ovals show magnifications of survivin-positive, late-mitotic cells. Quantification of SO is presented to the right of each image. (E) Same data as in (D), plotted as a cumulative frequency distribution. Not significant (*P* = 0.3364) by Kolmogorov–Smirnov test. (F) Quantification of epidermal thickness from the data shown in (B) and (G). Quantification of epidermal thickness in Ctrl, *shBcam-1554-*, *shBcam-1554*, and *Xiap-2297* is shown in Fig 2. Data are the mean ± SD of *n =* 40 microscopic fields from 4 embryos. ****P < 0.0001 for *shBcam-1554* vs. *shBcam-1554*;GFP-*Xiap*, **P = 1 × 10^−3^ for *control* vs. *shBcam-1554*;GFP-*Xiap*; not significant (*P* = 0.5242) for *shBcam-1554* mosaic tissue vs. *shBcam-1554*, ****P < 0.0001 for *shBcam-1554* mosaic tissue vs. control (G) Sagittal views of 10-μm sections of dorsal skin from *shScr-*, *shBcam-1554*;GFP-*Xiap-*, *shBcam*-*1554-*, *shBcam*-*1690-*, and *shXiap*-*2297*-transduced E16.5 embryos immunostained for the cleavage furrow marker survivin (red). H2B-GFP+ (green) and H2B-GFP− cells denote infected and uninfected cells, respectively. White circles indicate survivin-positive, late-mitotic, uninfected cells. Quantification of SO is presented to the right of each image. (H) Same data as in (G), plotted as a cumulative frequency distribution. E16.5, *P = 1.66 × 10^−2^ for control vs. *Bcam*-*1554*, *P = 2.7 × 10^−2^ for control vs. *Bcam*-*1690*, *P = 1.91 × 10^−2^ for control vs. *Xiap-2297*, and not significant for control vs. *shBcam-1554*;GFP-*Xiap* (*P* = 0.8455) by Kolmogorov–Smirnov test. The data underlying all the charts in the figure are included in S1 Data. Nuclei were stained with DAPI (blue). Dotted lines indicate the dermal–epidermal border. Scale bars = 20 μm. RT-PCR, reverse transcription PCR; SO, spindle orientation.

Having shown that BCAM inhibits apoptosis and is also required for normal SO and balanced symmetric/asymmetric cell division in the developing epidermis, we next asked whether apoptosis and SO might be linked. Given that overexpression of XIAP is known to inhibit apoptosis [58–60], we generated *shScr;GFP-Xiap* (XIAP overexpression) and *shBcam-1554*;GFP-*Xiap* (*Bcam* KD and XIAP overexpression) viruses and confirmed the ability of the *shBcam-1554*;GFP-*Xiap* virus to deplete *Bcam* with concomitant overexpress of *Xiap* in 1°MKs (Fig 3A). Next, we transduced E9 embryos with the *shScr;GFP-Xiap* virus and confirmed that XIAP overexpression does not alter the overall ratio between parallel and perpendicular SO (0°–45° and 45°–90°, respectively) (S6 Fig). In agreement with previous reports showing that *Xiap* overexpression can suppress apoptosis [58–60], GFP-*Xiap* overexpression reduced the elevated apoptosis observed in *Bcam*-depleted embryos to control levels (Fig 3B and 3C. Data from 3C is from Fig 1). Next, we examined SO in the same *Bcam* KD; *Xiap* overexpressing embryos by staining dorsal skin sections for survivin. Whereas *Bcam* depletion alone led to an increase in parallel divisions (Fig 2C), this increase was suppressed by *Xiap* overexpression (Fig 3D and 3E). Moreover, while the epidermal thickness of *Bcam* KD; *Xiap* overexpressing embryos was thinner than control embryos, it increased by approximately 20% compared to *shBcam*-transduced embryos (Fig 3F). Together, these data suggest that apoptosis and epidermal architecture influence SO in the developing epidermis.

### Apoptosis functions as an extrinsic cue that enhances symmetric cell division

Caspases have a variety of functions in nonapoptotic processes [61,62]; for instance, in the epidermis, caspase 3 cleaves α-catenin [47], which is essential for epidermal SO [19]. Therefore, to determine whether caspase 3 activation and apoptosis functions in an extrinsic fashion or may additionally play a cell-autonomous role in SO, we transduced embryos with *shScr*, *shBcam*, *shXiap*, or *shBcam*;GFP-*Xiap*, viruses and focus our attention on mosaic epidermal patches (i.e., approximately 50% transduction versus approximately 85% transduction in equivalent experiments shown in Fig 2). We first confirmed that apoptotic cells can be detected in *shBcam-1554*-transduced mosaic epidermal tissue (S8 Fig) and that the mosaic epidermal patches were thinner than the control epidermis (Fig 3F). Next, we analyzed SO in uninfected cells in the mosaic tissue of E16.5 embryos (i.e., H2B-GFP-negative wild-type cells versus H2B-GFP+ transduced cells in equivalent experiments shown in Fig 2). In *shScr*-transduced embryos, approximately equal numbers of uninfected cells were undergoing symmetric and asymmetric cell divisions, as expected (46% and 54%, respectively; Fig 3G and 3H). However, the proportion of uninfected cells undergoing symmetric cell divisions was markedly increased in the epidermis of *shBcam-1554-*, *shBcam-1690*-, and *shXiap*-transduced embryos (70%, 68%, and 71%, respectively; Fig 3G and 3H), indicating that the frequency of symmetric cell divisions had been enhanced in an extrinsic fashion by manipulations associated with increased apoptosis. Once again, concomitant *Xiap* overexpression in *Bcam*-depleted mosaic tissue resulted in near-normalization of the proportion of uninfected cells undergoing symmetric and asymmetric cell divisions (43% and 57%, respectively; Fig 3G and 3H), formally establishing a link between *Bcam*, apoptosis, tissue architecture, and cell division symmetry in the epidermis.

Collectively, the results presented here identify an essential role for BCAM in cell survival, which, in turn, impacts tissue thickness, SO, delamination, and epidermal morphogenesis. The results also identify a novel function for apoptosis and tissue architecture as an extrinsic cue that enhances symmetric cell division in the developing epidermis.

## Discussion

SO is a highly conserved process that is essential for the establishment and maintenance of a healthy epidermis [12,15,16,19,63]. During normal epidermal development and homeostasis, SO patterns change several times. Early in epidermal development, nearly all divisions are parallel to the BM; during the later stratification process, SO shifts toward perpendicular divisions; and in the adult skin, SO once again orients parallel to the BM [11,12,19,20,51,52,64]. Nevertheless, the upstream cues that orchestrate epidermal SO responses throughout life remain poorly understood.

The results of the present study establish an unexpected role for BCAM—and apoptosis—as regulators of epidermal morphogenesis. Notably, loss of BCAM expression in the epidermis did not affect BM organization, cell adhesion, or apicobasal polarity. Instead, BCAM was shown to be important for cell survival, and its loss resulted in apoptosis and thinning of the epidermis that functioned as extrinsic cues to alter SO and enhance symmetric cell division in neighboring cells. Notably, this behavior could be both rescued by inhibiting apoptosis and partially phenocopied by deletion of the antiapoptotic regulator XIAP. The physiological importance of this process was confirmed by demonstrating defects in tissue growth and epidermal thickness in embryos with *Bcam*-depleted epidermis.

An intriguing avenue of future research would be to investigate whether BCAM regulates the intrinsic or extrinsic apoptotic cascade. Given that the overexpression of XIAP was able to rescue the BCAM phenotype partially, it is tempting to speculate that BCAM functions via the intrinsic apoptotic cascade. However, it is key to note that although widely perceived to be two distinct apoptotic pathways, upon ligation of a death ligand and the induction of extrinsic downstream components of the BCL-2 (e.g., Bid/tBid), the intrinsic apoptotic pathway is also activated and mitochondrial outer membrane permeabilization (MOMP) initiated [48]. Similarly, caspase-3 is also known to function by cleaving what are considered “upstream” apoptotic components, even if activated and cleaved by the caspase-8 extrinsic initiator and not the caspase-9 intrinsic initiator [65]. Therefore, important work should be performed to examine the convergence of these pathways in this setting and distinguish between the possible apoptotic effects of BCAM.

Recent publications have shown that interphase cell shape [55], proliferation [15], and overall epidermal thickness [51] impact SO in the developing epidermis. Morrow and colleagues showed that SO responds to hypoproliferative and hyperproliferative conditions by mediating decreased and increased asymmetric cell division, respectively [14]. Our results showed that the decrease in epidermal growth and thickness resulting from ectopic apoptosis in the basal layer of *Bcam*-depleted epidermis correlates with increased symmetric cell division. Moreover, In *Xiap*-depleted epidermis that exhibits distinct growth properties that involve both ectopic apoptosis and hyperproliferation, the increase in symmetric cell division was moderate. Our data regarding epidermal architecture is also in agreement with Damen and colleagues, which showed that perpendicular cell divisions could be detected in the second phase of epidermal stratification when the epidermis is thick [51]. Box and colleagues showed that SO is guided by the long axis of the basal layer cell [55]. While we did not detect a defect in *Bcam*-depleted E16.5 basal layer cell shape, we cannot rule out the possibility that cell death, which occurs only in the basal layers of E15.5 and E16.5 *Bcam*-depleted epidermis, induces a transient cell elongation that impacts SO, as was demonstrated in the adult mouse skin [64]. An alternative explanation may involve the secretion of signals from apoptotic keratinocytes [66]. Indeed, Wnt ligands were shown to guide SO in cultured stem cells [67]. Additional work is required to determine the molecular mechanisms underlying the involvement of cell growth, shape, and tissue topography on SO.

The results of the current study demonstrate that BCAM is a crucial regulator of fundamental cellular processes that control tissue growth. It is noteworthy that altered expression/function of BCAM has been implicated in a variety of human cancers [37,68–72]. Moreover, BCAM is overexpressed in both basal cell and squamous cell carcinomas compared with normal skin cells [73], suggesting that a potential role for BCAM in these cancers warrants further investigation. However, the mechanisms by which BCAM functions in cancer are poorly understood. Given the known crucial role of apoptosis and SO in cancer development and progression [65,74], it is tempting to speculate that BCAM may link these two key processes not only physiologically during normal tissue development but also pathologically in certain disease conditions.

## Materials and methods

### Mice and primary mouse keratinocytes

All experimental protocols were approved by the Tel Aviv University Animal Care and Use Committee, confirmation number TAU-MD-IL-2206-162-4. Hsd:ICR (CD1) mice (Envigo) were used for all experiments. Epidermal keratinocytes were isolated as previously described [49]. Briefly, dorsal skin was removed from newborn mice and incubated with dispase (Sigma-Aldrich), and the epidermis was isolated and treated with trypsin (Biological Industries). Keratinocytes were plated on fibroblast feeder cells for 4 passages and then plated in tissue culture dishes without feeder cells.

### Lentiviruses

Lentiviruses were produced as previously described [33,53,75]. Briefly, lentiviral plasmids were generated by cloning oligonucleotides into pLKO.1-TRC (gift from David Root, Broad Institute, Cambridge, MA, USA; Addgene plasmid #10878) or LV-GFP (gift from Elaine Fuchs, Rockefeller University, New York, NY, USA; Addgene plasmid #25999) by digestion with EcoRI and AgeI, as described in the Genetic Perturbation Platform (GPP) website (http://portals.broadinstitute.org/gpp/public/resources/protocols). The XIAP-eGFP plasmid [76,77] was a gift from Sarit Larisch (University of Haifa, Haifa, Israel).

shRNA sequences were obtained from GPP (http://portals.broadinstitute.org/gpp/public/): *Bcam* (*1554*) construct #TRCN0000113604, target sequence 5′-GTCCTGTGAAGCGTCTAACAT-3′; *Bcam* (*1690*) construct #TRCN0000113602, target sequence 5′-GCTGCTTTCTATTGCATGAGA-3′; *Xiap* (*2297*) construct #TRCN0000112297, target sequence 5′-GCTTTAGGTGAAGGCGATAAA-3′.

### In utero lentivirus injection

Lentiviruses were injected into gestating mice as previously described [33]. Briefly, females at E9 were anesthetized with isoflurane, injected with pain killer, Rheumocam Veterinary 5 mg/ml according to the manufacturer instructions (Chanelle Pharma, Irland) and each embryo (up to 6 per litter) was injected with 0.4 to 1 μl of approximately 2 × 10^9^ colony-forming units (CFUs) of the appropriate lentiviruses. Controls were both uninfected littermates of *shBcam*-*1554*/*1690*;H2B-GFP lentivirus-injected embryos and *shScr*;H2B-GFP lentivirus-injected embryos. Pregnant mice were euthanized at E14.5, 15.5, or 16.5 with CO_2_.

In utero lentivirus injection infects approximately 60% to 70% of the dorsal skin epidermis [33]. For cell-autonomous studies, H2B-GFP+ (infected) cells were analyzed in patches in which >85% of the cells were infected. For non-cell-autonomous studies, H2B-GFP− (uninfected) cells were analyzed in skin patches in which <50% of the cells were infected.

### In vitro lentivirus infection of keratinocytes

1°MKs were generated as described above and infected as previously described [33]. Briefly, 1°MKs were plated at 10^5^ cells/well in 6-well plates and infected with 250 μl of approximately 10^7^ CFU lentiviruses (*shScr* or *shBcam*-*1554*/*1690* with a puromycin resistance gene) in the presence of 100 μg/ml Polybrene (Sigma-Aldrich) for 48 h. Cells were then treated with 3 μg/ml puromycin (Sigma-Aldrich) for 72 h to select for infected cells. Selected cells were cultured with 1.5 μg/ml puromycin for an additional 24 h and then used in experiments.

### Semiquantitative RT-PCR

RNA was extracted from samples using a Direct-zol RNA extraction kit (Zymo Research; R2060), and equal amounts of RNA were reverse transcribed using ProtoScript First Strand cDNA Synthesis Kit (New England Biolabs). Semiquantitative PCR was conducted using a StepOnePlus System (Thermo Fisher Scientific). Amplifications (40 cycles) were performed using the primers indicated below and cDNA template mixed with LightCycler DNA Master SYBR Green mix. The specificity of the reactions was determined by subsequent melting curve analysis. StepOnePlus software was used to adjust for background fluorescence. mRNA levels were quantified using the number of cycles needed to reach the crossing point according to the 2-delta CT method. Data are presented as mRNA levels of the gene of interest normalized to peptidylprolyl isomerase B (Ppib) mRNA levels. The primers were as follows: *Bcam* forward 5′-GCTGTCGGGCTACTCAGGT-3′ and reverse 5′-AGTCCAGGGCGACTTGCT-3′; *Xiap* forward 5′-TGCAAGAGCTGGATTTTATGC-3′ and reverse 5′-GGTCTTCACTTGGCTTCCAAT-3′; Ppib forward 5′-GTGAGCGCTTCCCAGATGAGA-3′ and reverse 5′-TGCCGGAGTCGACAATGATG-3′.

### Antibodies for western blot analysis and immunofluorescence microscopy

Antibodies against the following proteins were purchased and used as follows: GFP (Abcam, ab13970, 1:3,000), keratin 14 (K14) (BioLegend, PRB-155P, 1:1,000), keratin 10 (K10) (BioLegend, PRB-159P, 1:1,000), keratin 6 (K6) (BioLegend, PRB-169P, 1:1,000), keratin 5 (K5) (Acris, BP5006, 1:100), loricrin (BioLegend, Poly19051, 1:1,000), nidogen (Santa Cruz Biotechnology, sc-33706, 1:2,000), laminin γ1 (Abcam, ab80580, 1:500), laminin 332 (Abcam, ab14509, 1:500), β4 integrin (BD Biosciences, clone 346-11A, 1:400), β1 integrin (Milipore, clone 12G10, 1:100), active β1 integrin (BD Biosciences, clone 9EG7, 1:400), Bcam (R&D Systems AF8299, 1:500 for western blot and 1:300 for immunofluorescence), glyceraldehyde 3-phosphate dehydrogenase (GAPDH; Cell Signaling Technology, 5174, 1:1,000), survivin (Cell Signaling Technology, 2808, 1:500), active caspase 3 (Cell Signaling Technology, 9661, 1:500), Ki67 (Abcam, ab15580, 1:500), pHH3 (Abcam, ab10543, 1:500), LGN (a gift from Elaine Fuchs, Rockefeller University, New York, NY, USA, 1:4000), Par3 (Millipore, 07–330, 1:500), pericentrin (BioLegend, PRB-432C, 1:500), E-cadherin (Cell Signaling Technology, 3195, 1:500), and α-catenin (Sigma-Aldrich, C8114, 1:500).

Secondary antibodies were of the appropriate species/isotype reactivity conjugated to Alexa Fluor 488, Alexa Fluor 647, or Rhodamine Red-X (Jackson ImmunoResearch). Nuclei were labeled with 4′,6-diamidino-2-phenylindole (DAPI; Sigma-Aldrich).

### Immunofluorescence microscopy and western blotting

For immunofluorescence microscopy, embryos were embedded in OCT (Scigen), frozen, sectioned at 10 μM using a Leica CM1860 cryostat, and fixed in 4% formaldehyde for 10 min. Sections were then blocked with 0.3% Triton X-100, 1% bovine serum albumin, 5% normal donkey serum in phosphate-buffered saline, or in MOM Basic kit reagent (Vector Laboratories). Sections were incubated with primary antibodies (see above) overnight at 4°C and with secondary antibodies for 1 h at room temperature. For whole-mount immunofluorescence microscopy, embryos were fixed for 1 to 3 h in 4% formaldehyde, and the dorsal skin was removed mechanically and stained as described above.

For western blot analysis, cells were lysed with RIPA buffer (Sigma-Aldrich) and proteins were quantified using a BCA kit (Pierce). Samples of 5 to 20 μg protein were separated by 12% SDS-PAGE and transferred to nitrocellulose membranes. Membranes were blocked and incubated overnight at 4°C with primary antibodies to BCAM (1:1,000) and GAPDH (1:1,000) and then with horseradish peroxidase-conjugated antibodies (1,10,000 dilution in blocking solution) at room temperature for 1 h. Blots were developed using an Enhanced Chemiluminescence Detection Kit (Biological Industries) according to the manufacturer’s instructions. Images were obtained using a FUSION FX7 spectra imaging system.

### Confocal microscopy

Images were acquired using a Nikon C2+ laser-scanning confocal microscope with a 60×/1.4 oil objective or a 20×/0.75 air objective (Nikon). Images were recorded as 1,024 × 1,024 square pixels. RGB images were assembled in ImageJ software (imagej.nih.gov), and panels were labeled in Adobe Illustrator CC.

### EdU incorporation assay and quantification of cell proliferation

Quantification of cell proliferation was performed as previously described [54,75]. Briefly, pregnant females were injected with the appropriate lentiviruses on E9 as described above. On E16.5, mice were injected with 25 mg/kg body weight of EdU for 2 h, after which the embryos were collected, frozen in OCT, sectioned (10-μm thick), and fixed in 4% PFA. Sections were incubated for 30 min in a solution containing copper sulfate, sulfo-cyanine 3 azide (2 μM; Lumiprobe D1330), and sodium ascorbate (100 mM; Acros 352685000). Sections were imaged by confocal fluorescence microscopy, and the number of GFP+ and EdU+ cells was counted. The percentage proliferating cells was calculated as (number of EdU+GFP+ double-positive cells/total number of GFP+ cells) × 100.

### Quantification of SO

SO was measured as previously described [12,53]. Briefly, embryos were injected with lentiviruses encoding *shScr*, *shBcam*, *shBcam;GFP-Xiap*, or *shXiap* on E9 and harvested at E16.5. Embryos were frozen in OCT, sectioned (10 μm), fixed, and incubated with anti-survivin antibody (1:500) overnight, followed by secondary antibody at room temperature for 1 h. Images were collected using a Nikon C2+/60×/1.4 objective and the angle between the two daughter nuclei and the BM was calculated using the “angle” tool in ImageJ.

### Quantification of epidermal thickness

Embryos of pregnant females were injected with lentiviruses encoding *shScr* or *shBcam* on E9 and harvested at E16.5. Embryos were frozen in OCT, sectioned (10 μm), fixed, and stained for K6 (1:1,000) and nidogen (1:2,000) overnight at 4°C, followed by secondary antibody at room temperature for 1 h. K6 and nidogen staining was imaged using a Nikon C2+/60×/1.4 objective to generate optical sections of 0.49 μm. Skin thickness was analyzed through the back skin interfollicular epidermis. The distance between the K6+ periderm and the BM was measured using the “freehand lines” tool in ImageJ.

### Axial ratio calculation

To quantify the axial ratio of early mitotic cells, whole-mount samples were immunostained for E-cadherin and confocal images were collected at a single plane through the middle of the basal layer. Early mitotic cells were identified by DAPI staining and the axial ratio was calculated using the “fit ellipse” tool in ImageJ.

### Quantification of cell shape

To measure cell height and width, 10 μm sagittal sections of dorsal skin were stained for E-cadherin and imaged with confocal microscopy. Cell height and width were measured using the line tool in ImageJ.

### Senescence-associated β-galactosidase assay

Senescence-associated β-galactosidase assay was done as previously described [33,46]. Briefly, embryos were injected with lentiviruses harboring scramble *shScr* or *shBcam* on E9 and harvested at E16.5. Embryos were frozen in OCT, sectioned (10 μm), fixed in 0.5% glutaraldehyde for 15 min, stained overnight at 37°C with 40 mmol/L phosphate buffer (pH = 6) with 5 mmol/L K_4_Fe(CN)_6_, 5 mmol/L K_3_Fe(CN)_6_, 150 mmol/L NaCl, 2 mmol/L MgCl_2_, and 1 mg/mL X-gal, washed in PBS, fixed in 95% ethanol for 15 min, counterstained with nuclear fast red, dehydrated, and mounted.

### Adherens junction assembly assay

Control and *Bcam*-depleted 1°MK cells were seeded in 24-well plates in low-calcium medium (50 μM) at high confluency (8 × 104 cells/well). Upon formation of a confluent monolayer, the medium was switched to high-calcium medium (1.5 mM) and the cells were incubated for an additional 24 h. The cells were then fixed in paraformaldehyde and stained for E- cadherin.

### Quantification of focal adhesion (FA) parameters

Control and *Bcam*-depleted 1°MK were seeded in 24-well plates (10,000 cells/well) in low-calcium media (50 μM Ca2+) 24 h later. Cells were fixed in paraformaldehyde and stained for β1 integrin, 9EG7 epitope, and paxillin. Images were acquired using a Nikon C2+ laser-scanning confocal microscope using a 60×/1.4 objective. For FA analyses, paxillin staining was automatically segmented using the adaptive threshold plug-in of Image J. Segmented FAs were analyzed using analyze particles option of Image J. FAs with an area of 1 to 10 μm^2^ area were considered for the analysis.

### Statistical analysis

Quantitative data are shown as the mean ± SD unless noted. Analyses were performed using Prism (GraphPad). Sample sizes and the specific tests performed are indicated in the figure legends. No statistical method was used to predetermine the sample size. Experiments were not randomized, and investigators were not blinded to sample identity during experiments or outcome assessments.

## Supporting information

S1 Fig Distribution of β1 integrin and basement membrane proteins in the epidermis is unaffected by *Bcam* depletion. (A) Sagittal views of 10-μm sections of dorsal skin from control and *Bcam*-*1554* KD E16.5 embryos immunostained for total β1 integrin (white) or active β1 integrin (9EG7 epitope; white). (B) Dorsal skin sections from embryos treated as in (A) and immunostained for the basement membrane proteins laminin γ1, laminin 332, and nidogen (white). Nuclei were stained with DAPI (blue), and upper right insets show the transduced cells (H2B-GFP+). Scale bars = 20 μm.
(TIF)

S2 Fig Normal adhesion and differentiation in *Bcam*-depleted cultured keratinocytes. (A) *shScr*- (Ctrl) and *shBcam 1554-*transduced primary mouse keratinocytes were cultured in low-calcium (50 μM) media and then immunolabeled for β1 integrin and active β1 integrin (9EG7 epitope). Quantification of β1 integrin level is presented to the right of each image. Data are the mean ± SD of *n =* 64 Ctrl cells and 65 *shBcam 1554* cells from 3 experiments. β1 integrin, not significant for control vs. *Bcam*-*1554* (*P* = 0.127); active β1 integrin (9EG7 epitope), **P = 2.8 × 10^−3^ for control vs. *Bcam*-*1554* by unpaired *t* test. (B) *shScr*- (Ctrl) and *shBcam 1554*-transduced primary mouse keratinocytes were cultured in low-calcium (50 μM) media and immunolabeled for paxillin. (C) Quantification of focal adhesion number and area from data shown in (B). Data are the mean ± SD of *n* = 41 cells from 2 experiments. Focal adhesion number, not significant for control vs. *Bcam*-*1554* (*P* = 0.6814); focal adhesion area, ***P = 2 × 10^−4^ for control vs. *Bcam*-*1554*. (D) *shScr*- (Ctrl) and *shBcam 1554*-transduced primary mouse keratinocytes were induced to form adherens junctions by switching from low-calcium (50 μM) to high-calcium (1.5 mM) media and then immunolabeled for E-cadherin at the indicated time points. (E) Western blot analysis of *shScr*- (Ctrl) and *shBcam 1554*-transduced primary mouse keratinocytes grown in high-calcium media. Blots were probed with antibodies to K14, K10, or GAPDH (loading control). The data underlying all the charts in the figure are included in S1 Data. Nuclei were stained with DAPI (blue). Scale bars = 20 μm.
(TIF)

S3 Fig Normal differentiation, apoptosis, thickness, and adhesion in E14.5 *Bcam*-depleted epidermis. **(**A) Sagittal views of 10-μm sections of dorsal skin from control and *Bcam*-*1554* KD E14.5 embryos immunostained for the basal layer marker keratin 14 and suprabasal layer maker keratin 10 (red). (B) Dorsal skin sections from embryos treated as in (A) and immunostained for active caspase 3 (red). (C) Dorsal skin sections from embryos treated as in (A) and coimmunostained for keratin 6 (red) and nidogen (white). (D) Dorsal skin sections from embryos treated as in (A) and immunostained for the adherens junction proteins E-cadherin (left) and α-catenin (right). Nuclei were stained with DAPI (blue). Dotted lines indicate the dermal–epidermal border, and upper right insets show the transduced cells (H2B-GFP+). Scale bars = 20 μm.
(TIF)

S4 Fig Defects in apoptosis, cell delamination, and epidermal thickness in E15.5 *Bcam*-depleted epidermis. (A) Sagittal views of 10-μm sections of dorsal skin from control and *Bcam*-*1554* KD E15.5 embryos immunostained for the basal layer marker keratin 14, suprabasal layer maker keratin 10, and the granular layer marker loricrin (red). (B) Dorsal skin sections from embryos treated as in (A) and immunostained for active caspase 3 (red). (C) Dorsal skin sections from embryos treated as in (A) and coimmunostained for keratin 14 (red) and keratin 10 (white) white circles indicate double positive basal layer cells. (D) Dorsal skin sections from embryos treated as in (A) and immunostained for the cleavage furrow marker survivin (red). White circles indicate survivin+ cells. (E) Dorsal skin sections from embryos treated as in (A) and coimmunostained for keratin 6 (red) and nidogen (white). (F) Dorsal skin sections from embryos treated as in (A) and immunostained for the adherens junction proteins E-cadherin (left) and α-catenin(right). Nuclei were stained with DAPI (blue). Dotted lines indicate the dermal–epidermal border, and upper right insets show the transduced cells (H2B-GFP+). Scale bars = 20 μm.
(TIF)

S5 Fig Normal differentiation, senescence, adhesion, and polarity in *Bcam*-depleted E16.5 epidermis. (A) Sagittal views of 10-μm sections of dorsal skin from control and *Bcam*-*1554* KD E16.5 embryos immunostained for the basal layer marker keratin 14, suprabasal layer maker keratin 10, and the granular layer marker filaggrin (red). Nuclei were stained with DAPI. Dotted lines indicate the dermal–epidermal border, and upper right insets show the transduced cells (H2B-GFP+). (B) Dorsal skin sections from embryos treated as in (A) and immunohistochemically stained for senescence-associated β-galactosidase. (C) Dorsal skin sections from embryos treated as in (A) and immunostained for the adherens junction proteins E-cadherin (left) and α-catenin(right). (D) Dorsal skin sections from embryos treated as in (A) and immunostained for the polarity proteins Par3 (left) and pericentrin(right).
(TIF)

S6 Fig XIAP overexpression and depletion in the dorsal skin of E16.5 embryos. (A) Sagittal views of 10-μm sections of dorsal skin from *shXiap*-2297-transduced E16.5 embryos immunostained for the cleavage furrow marker survivin (red). White circles indicate survivin-positive, late-mitotic cell. (B) Sagittal views of 10-μm sections of dorsal skin from *shScr*;GFP-*Xiap*-transduced E16.5 embryos immunostained for the cleavage furrow marker survivin (red). White circles indicate survivin-positive, late-mitotic, uninfected cells. Quantification of spindle orientation is presented to the right of the image. (C) Same data as in (B), plotted as a cumulative frequency distribution. Not significant (*P* = 0.0977) by Kolmogorov–Smirnov test. The data underlying all the charts in the figure are included in S1 Data. Nuclei were stained with DAPI (blue). Dotted lines indicate the dermal–epidermal border. Scale bars = 20 μm.
(TIF)

S7 Fig Cell shape in the epidermis is unaffected by *Bcam* depletion. (A) Sagittal views of 10-μm sections of dorsal skin from control and *Bcam*-*1554* KD E16.5 embryos immunostained for E-cadherin (red). (B) Quantification of basal layer cell width and height from the data shown in (A). *N =* 172 and 173 cells for control and *Bcam*-*1554*-transduced cells, respectively, from 3 embryos per condition. Horizontal bars represent the mean and SEM, and circles represent individual cells. *P* = 0.0621 and *P* = 0.1194 for cell width and height, respectively, by unpaired two-tailed *t* test. (C) Whole-mount immunofluorescence images from embryos treated as in (A). (D) Quantification of mitotic cell axial ratio from the data shown in (C). *N* = 45 and 43 control and *Bcam*-*1554*-transduced cells, respectively, from 4 embryos per condition. Horizontal bars represent the mean and SEM and circles represent individual cells. Not significant (*P* = 0.0664) by unpaired *t* test. The data underlying all the charts in the figure are included in S1 Data.
(TIF)

S8 Fig Defects in apoptosis in mosaic E16.5 *Bcam*-depleted epidermis. Sagittal view of 10-μm sections of dorsal skin from mosaic *Bcam*-*1554* KD E16.5 embryos immunostained for the apoptosis marker active caspase 3 (red). Nuclei were stained with DAPI (blue). Dotted lines indicate the dermal–epidermal border. Scale bars = 20 μm.
(TIF)

S1 Data Numerical data were used for charts and statistical analysis. (XLSX)

S1 Raw images Western blot membrane images. (TIFF)

## Abbreviations

BCAM: basal cell adhesion molecule
BM: basement membrane
CFU: colony-forming unit
H2B-GFP: GFP-tagged histone 2B reporter
KD: knockdown
MOMP: mitochondrial outer membrane permeabilization
shRNA: short hairpin RNA
shScr: control scrambled shRNA
SO: spindle orientation
