# Supplementary figures and images for: Apoptosis and tissue thinning contribute to symmetric cell division in the developing mouse epidermis in a nonautonomous way
Source: PLoS Biol. 2022 Aug 15;20(8):e3001756. doi: 10.1371/journal.pbio.3001756 (PMC9410552; doi:10.1371/journal.pbio.3001756)

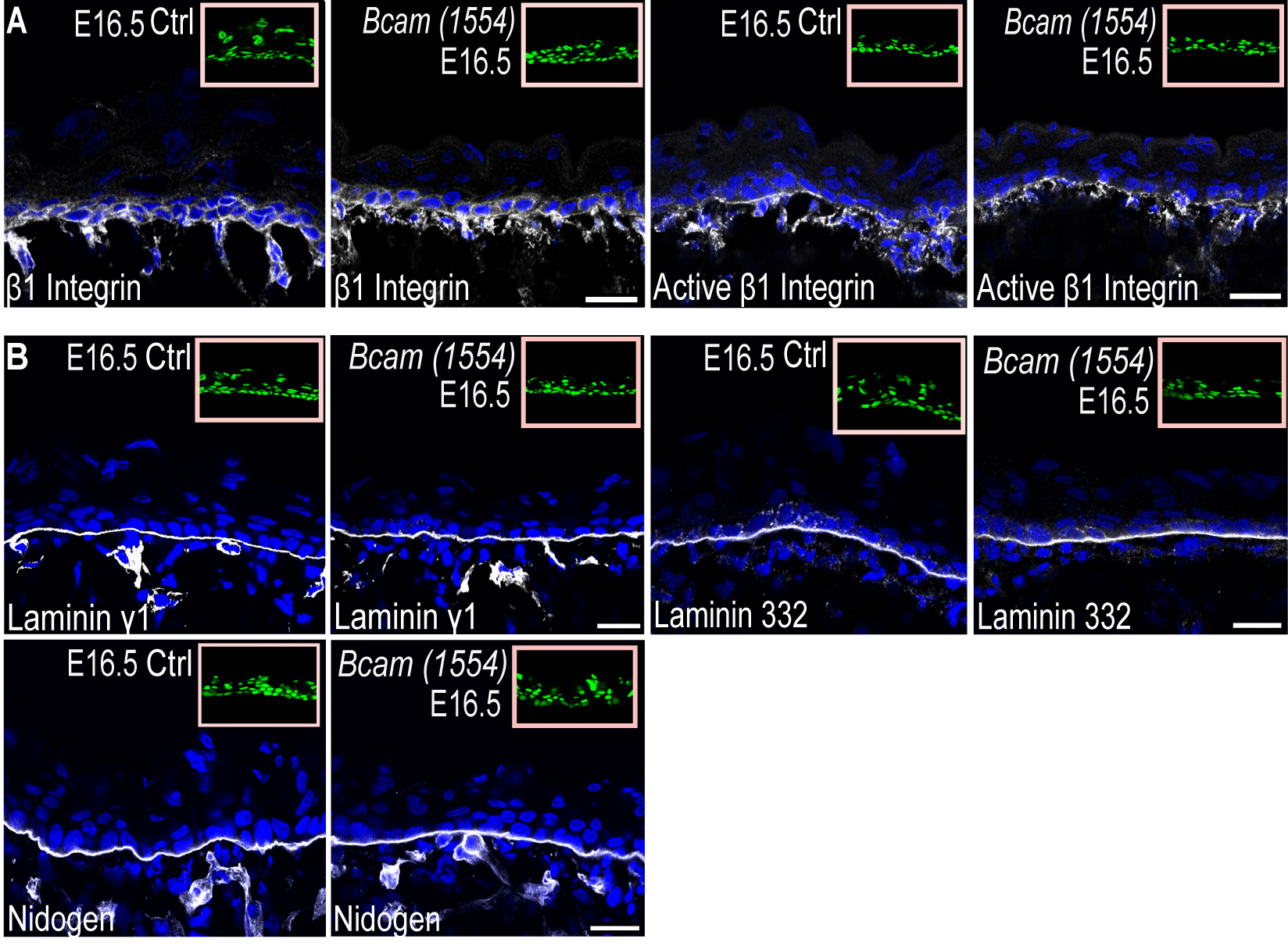

Supplement: S1 Fig — (A) Sagittal views of 10-μm sections of dorsal skin from control and Bcam-1554 KD E16.5 embryos immunostained for total β1 integrin (white) or active β1 integrin (9EG7 epitope; white). (B) Dorsal skin sections from embryos treated as in (A) and immunostained for the basement membrane proteins laminin γ1, laminin 332, and nidogen (white). Nuclei were stained with DAPI (blue), and upper right insets show the transduced cells (H2B-GFP+). Scale bars = 20 μm. (TIF) [file pbio.3001756.s001.tif]

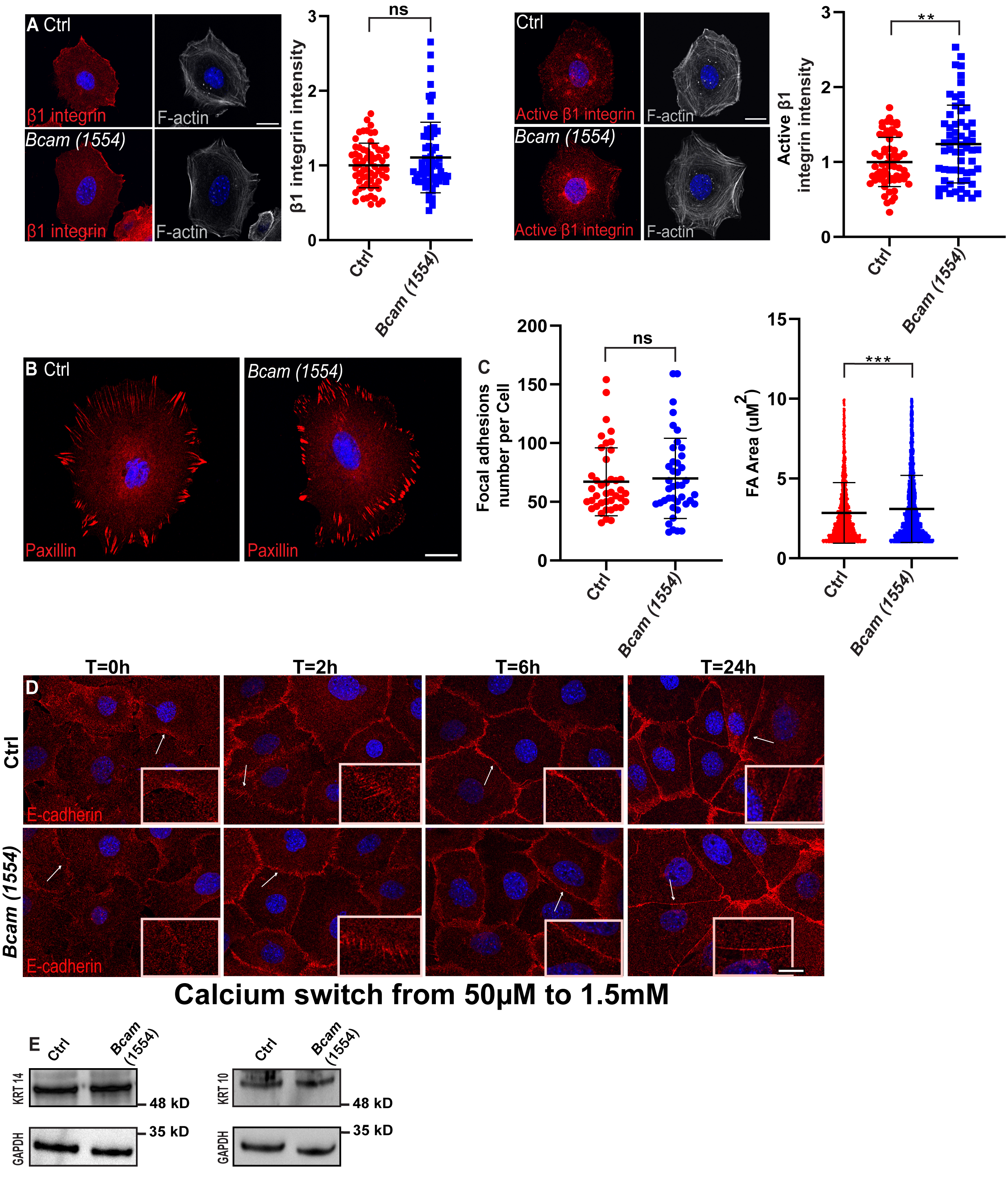

Supplement: S2 Fig — (A) shScr- (Ctrl) and shBcam 1554-transduced primary mouse keratinocytes were cultured in low-calcium (50 μM) media and then immunolabeled for β1 integrin and active β1 integrin (9EG7 epitope). Quantification of β1 integrin level is presented to the right of each image. Data are the mean ± SD of n = 64 Ctrl cells and 65 shBcam 1554 cells from 3 experiments. β1 integrin, not significant for control vs. Bcam-1554 (P = 0.127); active β1 integrin (9EG7 epitope), **P = 2.8 × 10^−3^ for control vs. Bcam-1554 by unpaired t test. (B) shScr- (Ctrl) and shBcam 1554-transduced primary mouse keratinocytes were cultured in low-calcium (50 μM) media and immunolabeled for paxillin. (C) Quantification of focal adhesion number and area from data shown in (B). Data are the mean ± SD of n = 41 cells from 2 experiments. Focal adhesion number, not significant for control vs. Bcam-1554 (P = 0.6814); focal adhesion area, ***P = 2 × 10^−4^ for control vs. Bcam-1554. (D) shScr- (Ctrl) and shBcam 1554-transduced primary mouse keratinocytes were induced to form adherens junctions by switching from low-calcium (50 μM) to high-calcium (1.5 mM) media and then immunolabeled for E-cadherin at the indicated time points. (E) Western blot analysis of shScr- (Ctrl) and shBcam 1554-transduced primary mouse keratinocytes grown in high-calcium media. Blots were probed with antibodies to K14, K10, or GAPDH (loading control). The data underlying all the charts in the figure are included in S1 Data. Nuclei were stained with DAPI (blue). Scale bars = 20 μm. (TIF) [file pbio.3001756.s002.tif]

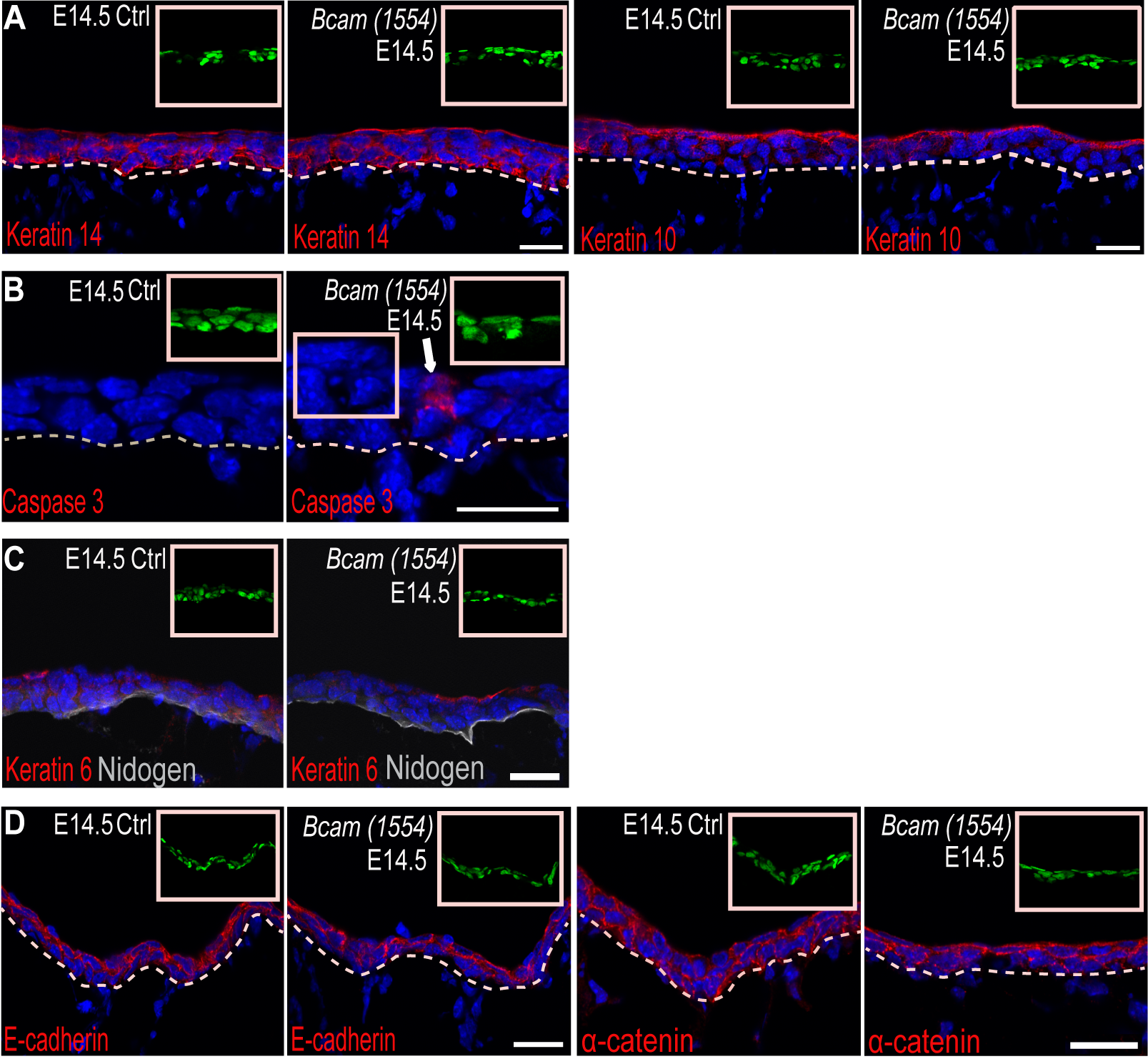

Supplement: S3 Fig — (A) Sagittal views of 10-μm sections of dorsal skin from control and Bcam-1554 KD E14.5 embryos immunostained for the basal layer marker keratin 14 and suprabasal layer maker keratin 10 (red). (B) Dorsal skin sections from embryos treated as in (A) and immunostained for active caspase 3 (red). (C) Dorsal skin sections from embryos treated as in (A) and coimmunostained for keratin 6 (red) and nidogen (white). (D) Dorsal skin sections from embryos treated as in (A) and immunostained for the adherens junction proteins E-cadherin (left) and α-catenin (right). Nuclei were stained with DAPI (blue). Dotted lines indicate the dermal–epidermal border, and upper right insets show the transduced cells (H2B-GFP+). Scale bars = 20 μm. (TIF) [file pbio.3001756.s003.tif]

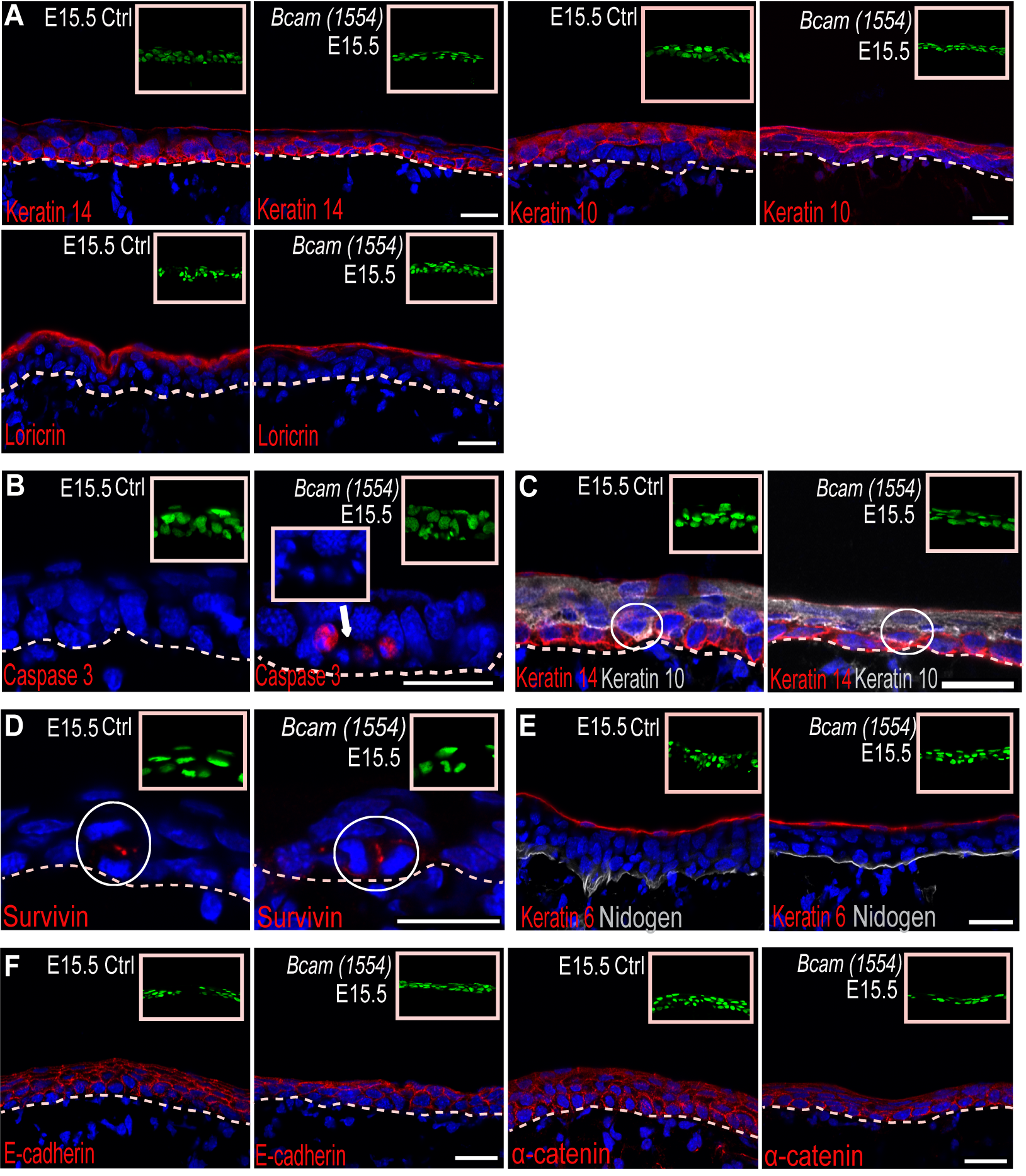

Supplement: S4 Fig — (A) Sagittal views of 10-μm sections of dorsal skin from control and Bcam-1554 KD E15.5 embryos immunostained for the basal layer marker keratin 14, suprabasal layer maker keratin 10, and the granular layer marker loricrin (red). (B) Dorsal skin sections from embryos treated as in (A) and immunostained for active caspase 3 (red). (C) Dorsal skin sections from embryos treated as in (A) and coimmunostained for keratin 14 (red) and keratin 10 (white) white circles indicate double positive basal layer cells. (D) Dorsal skin sections from embryos treated as in (A) and immunostained for the cleavage furrow marker survivin (red). White circles indicate survivin+ cells. (E) Dorsal skin sections from embryos treated as in (A) and coimmunostained for keratin 6 (red) and nidogen (white). (F) Dorsal skin sections from embryos treated as in (A) and immunostained for the adherens junction proteins E-cadherin (left) and α-catenin(right). Nuclei were stained with DAPI (blue). Dotted lines indicate the dermal–epidermal border, and upper right insets show the transduced cells (H2B-GFP+). Scale bars = 20 μm. (TIF) [file pbio.3001756.s004.tif]

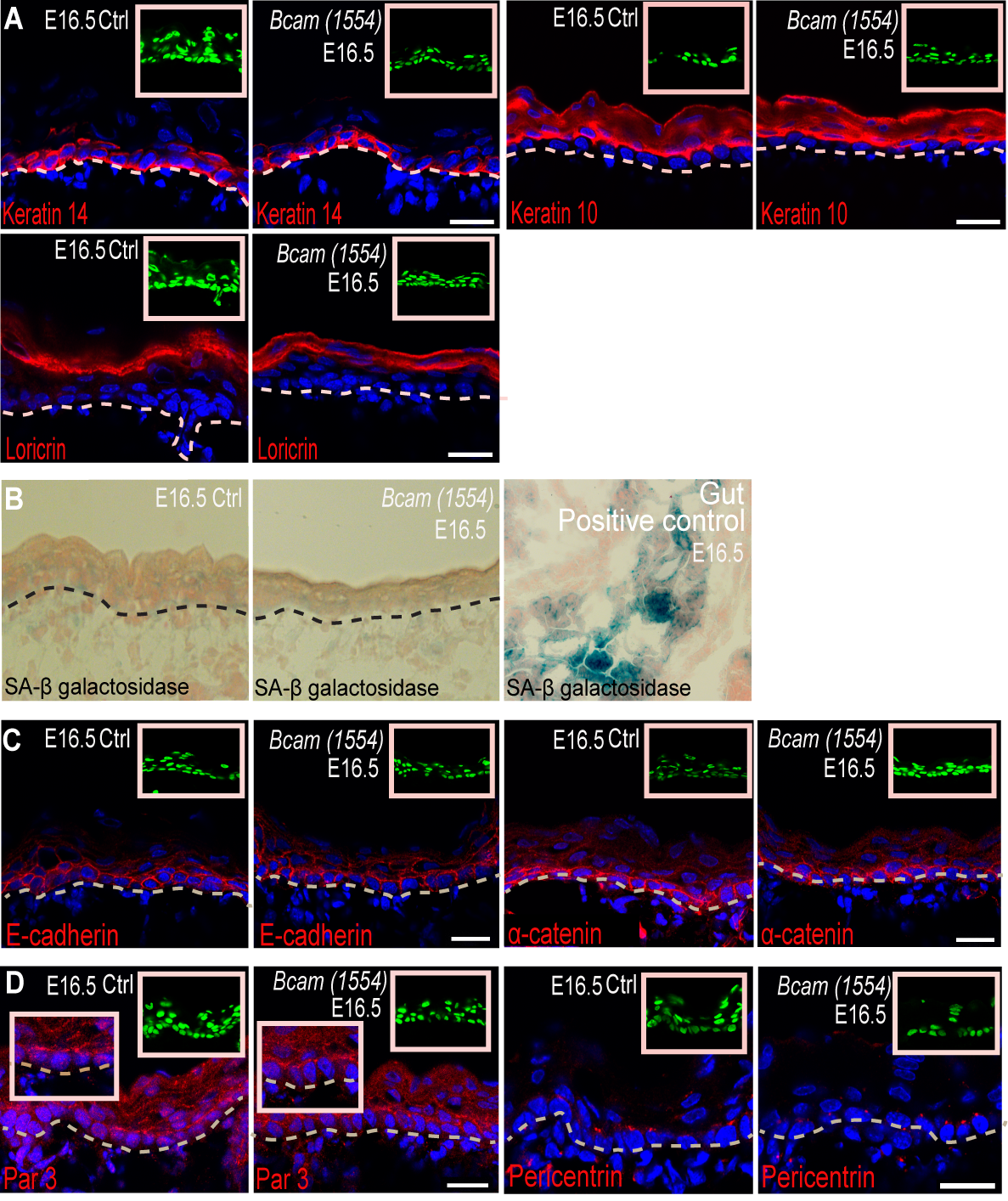

Supplement: S5 Fig — (A) Sagittal views of 10-μm sections of dorsal skin from control and Bcam-1554 KD E16.5 embryos immunostained for the basal layer marker keratin 14, suprabasal layer maker keratin 10, and the granular layer marker filaggrin (red). Nuclei were stained with DAPI. Dotted lines indicate the dermal–epidermal border, and upper right insets show the transduced cells (H2B-GFP+). (B) Dorsal skin sections from embryos treated as in (A) and immunohistochemically stained for senescence-associated β-galactosidase. (C) Dorsal skin sections from embryos treated as in (A) and immunostained for the adherens junction proteins E-cadherin (left) and α-catenin(right). (D) Dorsal skin sections from embryos treated as in (A) and immunostained for the polarity proteins Par3 (left) and pericentrin(right). (TIF) [file pbio.3001756.s005.tif]

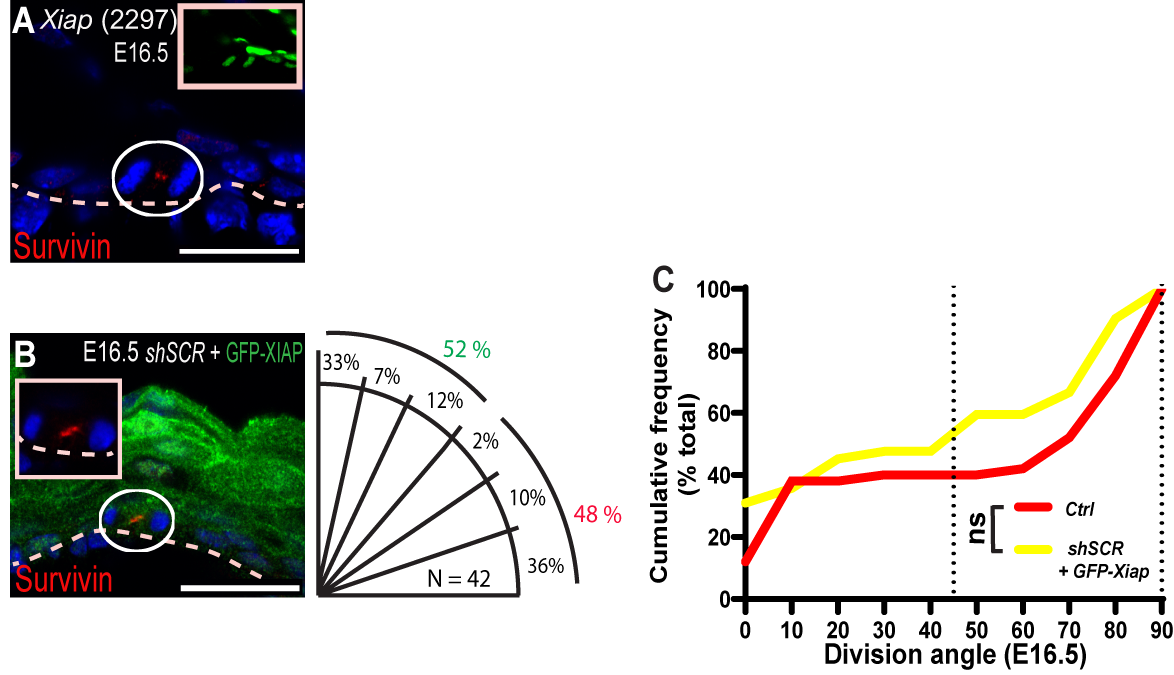

Supplement: S6 Fig — (A) Sagittal views of 10-μm sections of dorsal skin from shXiap-2297-transduced E16.5 embryos immunostained for the cleavage furrow marker survivin (red). White circles indicate survivin-positive, late-mitotic cell. (B) Sagittal views of 10-μm sections of dorsal skin from shScr;GFP-Xiap-transduced E16.5 embryos immunostained for the cleavage furrow marker survivin (red). White circles indicate survivin-positive, late-mitotic, uninfected cells. Quantification of spindle orientation is presented to the right of the image. (C) Same data as in (B), plotted as a cumulative frequency distribution. Not significant (P = 0.0977) by Kolmogorov–Smirnov test. The data underlying all the charts in the figure are included in S1 Data. Nuclei were stained with DAPI (blue). Dotted lines indicate the dermal–epidermal border. Scale bars = 20 μm. (TIF) [file pbio.3001756.s006.tif]

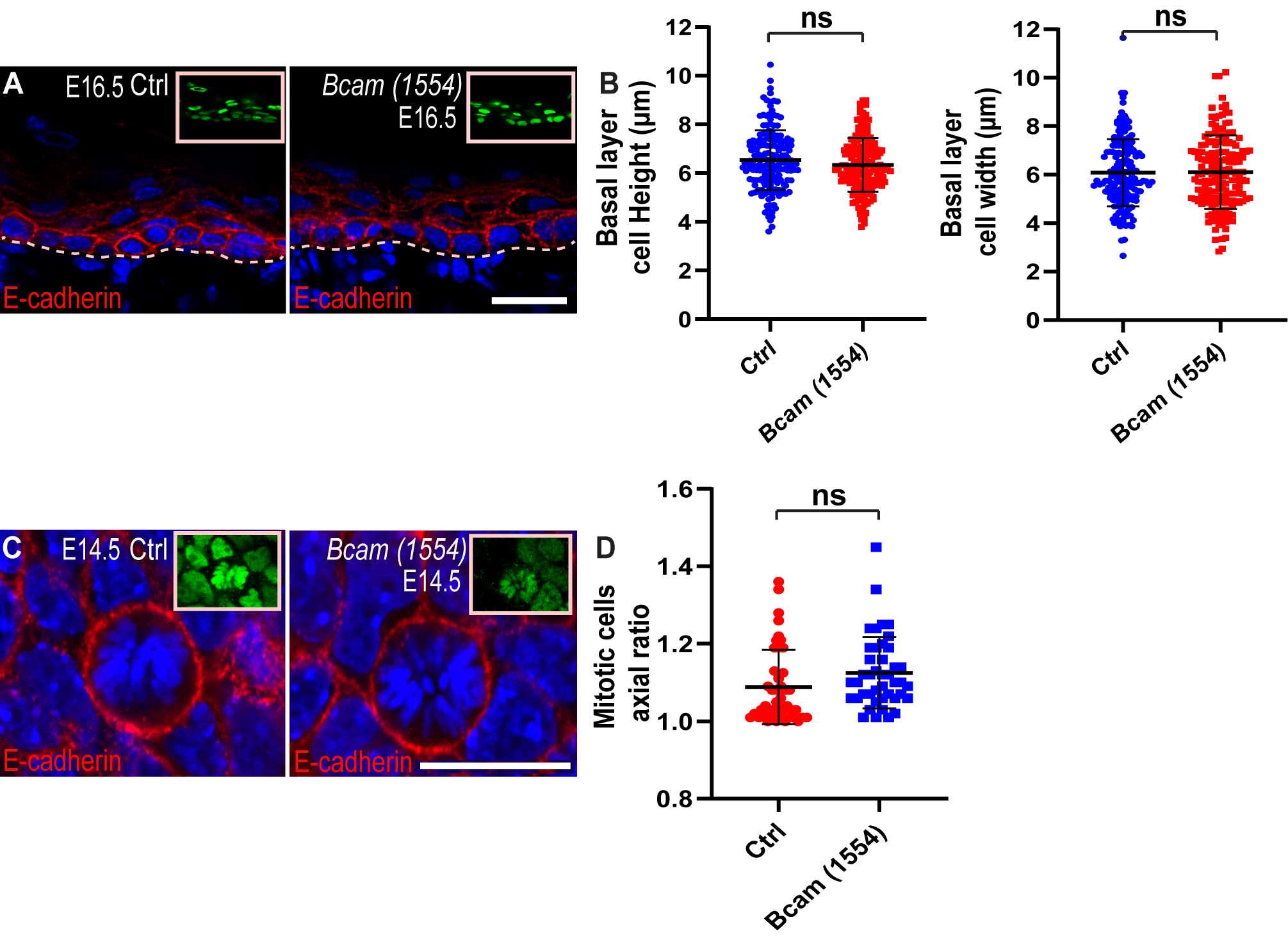

Supplement: S7 Fig — (A) Sagittal views of 10-μm sections of dorsal skin from control and Bcam-1554 KD E16.5 embryos immunostained for E-cadherin (red). (B) Quantification of basal layer cell width and height from the data shown in (A). N = 172 and 173 cells for control and Bcam-1554-transduced cells, respectively, from 3 embryos per condition. Horizontal bars represent the mean and SEM, and circles represent individual cells. P = 0.0621 and P = 0.1194 for cell width and height, respectively, by unpaired two-tailed t test. (C) Whole-mount immunofluorescence images from embryos treated as in (A). (D) Quantification of mitotic cell axial ratio from the data shown in (C). N = 45 and 43 control and Bcam-1554-transduced cells, respectively, from 4 embryos per condition. Horizontal bars represent the mean and SEM and circles represent individual cells. Not significant (P = 0.0664) by unpaired t test. The data underlying all the charts in the figure are included in S1 Data. (TIF) [file pbio.3001756.s007.tif]

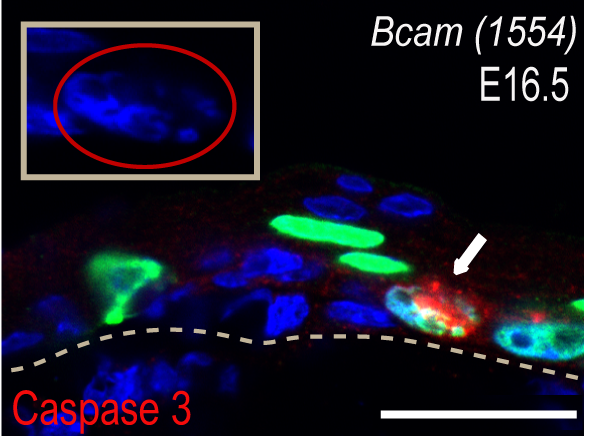

Supplement: S8 Fig — Sagittal view of 10-μm sections of dorsal skin from mosaic Bcam-1554 KD E16.5 embryos immunostained for the apoptosis marker active caspase 3 (red). Nuclei were stained with DAPI (blue). Dotted lines indicate the dermal–epidermal border. Scale bars = 20 μm. (TIF) [file pbio.3001756.s008.tif]

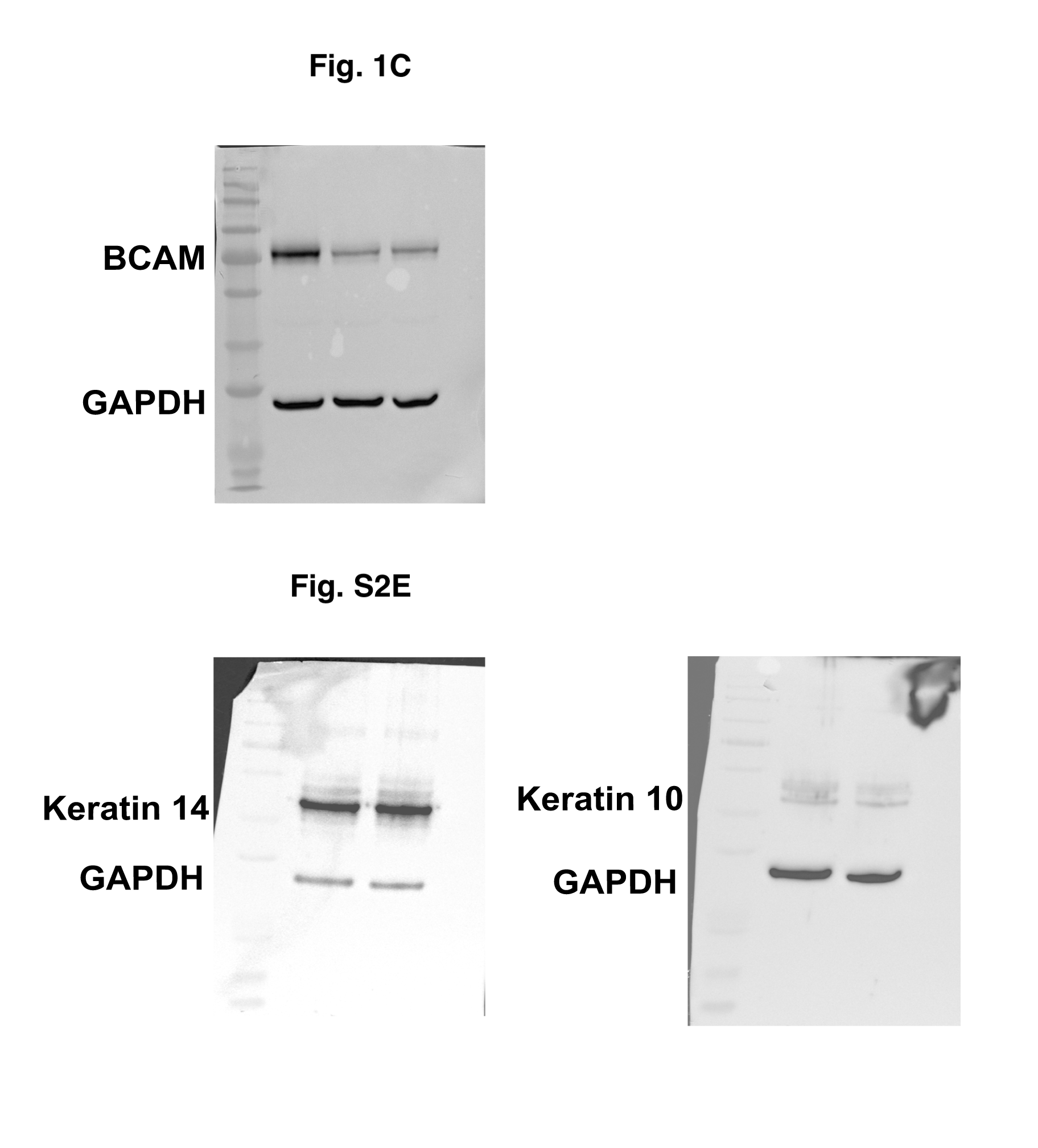

Supplement: S1 Raw images — (TIFF) [file pbio.3001756.s010.tiff]
